# Computational identification of novel therapeutic candidates for *Streptococcus pyogenes* and influenza A coinfections through transcriptomic-based drug repositioning

**DOI:** 10.1186/s12866-026-05063-y

**Published:** 2026-04-23

**Authors:** Kevin Strey, Salem Sueto, Georg Fuellen, Bernd Kreikemeyer, Nadja Patenge, Israel Barrantes

**Affiliations:** 1 Institute of Medical Microbiology, Virology and Hygiene (IMIKRO), Rostock University Medical Center, Rostock, Germany; 2 Institute for Biostatistics and Informatics in Medicine and Ageing Research (IBIMA), Rostock University Medical Center, Rostock, Germany

**Keywords:** Dual RNA-seq, *Streptococcus pyogenes*, Influenza A virus, coinfection, drug repurposing

## Abstract

**Background:**

Influenza A virus (IAV) causes severe illness with a high mortality rate, and secondary bacterial infections can lead to severe pneumonia. Despite the availability of antibiotics and antivirals, treatment of concurrent IAV and invasive group A streptococcal infections remains challenging. As bioinformatic drug repurposing represents a cost- and time-effective manner for discovering novel treatments, this study aimed at the identification of novel therapeutic options for the *S. pyogenes*-IAV coinfection through this computational approach.

**Results:**

Following in vitro infections of pharyngeal epithelial cell lines with either IAV or *S. pyogenes* serotypes M1 or M49, transcriptomic changes in host cells were analyzed by RNA-seq, obtaining patterns of differentially expressed genes for each infection. These genes were then queried against the LINCS L1000 small molecule database to find compounds capable of reversing infection-induced molecular phenotypes. In this manner, we identified through computational analyses the antitumoral and antibacterial compound mitoxantrone as well as three kinase inhibitors: AT7519 and flavopiridol, which act against cyclin-dependent kinases, and BI2536, an inhibitor of the Polo-like kinase 1 (PLK1), as the main candidates to treat these coinfections.

**Conclusions:**

Using computational drug repurposing we identified four compounds that have the potential to act as suitable drugs in IAV-*S. pyogenes* coinfections. BI2536 and flavopiridol have been previously confirmed as active against IAV infections in vitro, while mitoxantrone is effective against *S. pneumoniae*. These results validate our approach, which offers a cost-effective alternative to large-scale drug screenings to find suitable candidate compounds.

**Supplementary Information:**

The online version contains supplementary material available at 10.1186/s12866-026-05063-y.

## Background

Influenza A viruses (IAVs) belong to the family of Orthomyxoviridae and are enveloped negative-strand RNA viruses. Their genome contains eight segments encoding at least 11 open reading frames [1]. IAVs are classified into different subtypes based on the two viral glycoproteins hemagglutinin (HA) and neuraminidase (NA). HA and NA are located in the outer envelope of the virus particle. Based on the antigenic diversity of HA and NA, which are two major immunogenic proteins, influenza viruses are classified into 18 HA subtypes (H1-H18) and 11 NA subtypes (N1-N11), respectively [2]. The natural reservoirs of IAV are wild avian species. Especially aquatic birds, e.g. ducks, geese, swans, and gulls, have been reported to carry IAV [3]. Host switch events to domestic poultry, horses, swine, dogs and humans occur frequently. Stable host switching leads to adaptation and the resulting viral lineages are transmissible in the new host [4]. Currently, IAV (H1N1) and IAV (H3N2) transmission in humans is established, but avian influenza viruses from subtypes H5, H7, and H9 have crossed the species barrier and caused sporadic human infections [5]. Typically, IAV causes a common seasonal disease with mild respiratory manifestation. However, among high risk groups, including pregnant women, the elderly, children and patients with immunosuppressive diseases, influenza infection is associated with high mortality. It is estimated by the World Health Organization (WHO) that these annual outbreaks result worldwide in about 3 to 5 million cases of severe illness, and about 290,000 to 650,000 respiratory deaths [6]. In addition to annual epidemics, four IAV pandemics were recorded in the past 110 years: 1918 H1N1 pandemic (also known as the Spanish flu), 1957–1958 H2N2 pandemic (Asian flu), 1968–1969 H3N2 pandemic (Hong Kong flu), and another H1N1 pandemic in 2009 (Mexican or swine flu) [7–10]. In the 1918 pandemic, approximately 50–100 million were killed globally [9]. IAV pathogenicity depends on viral virulence, host response, and secondary bacterial co-infections [11]. Secondary bacterial pneumonia has been shown to be the leading cause of viral-associated mortality during IAV pandemics [12]. *Streptococcus pneumoniae*, *Streptococcus pyogenes*, and *Haemophilus influenzae* were the most frequently observed bacteria in 1918 pandemic co-infections [13, 14].

*S. pyogenes* is a strictly human pathogen causing highly prevalent conditions, e.g. pharyngitis in children. Untreated infections frequently develop into severe complications associated with high mortality rates. A number of 18.1 million serious disease cases was estimated per year, accounting for 517,000 deaths [15]. The majority of deaths are caused by rheumatic heart disease, invasive streptococcal group A disease, and post-streptococcal glomerulonephritis [16]. The global burden of severe streptococcal diseases is rising, especially in low-resource settings [17]. Currently, no vaccine for streptococcal group A infections is available. *S. pyogenes* remains generally susceptible to penicillin, but alternative antibiotics are required in the case of adverse reactions or penicillin treatment failure. Occurrence of resistance to non-β‑lactam antibiotics, e.g. to macrolides, is dependent on the frequency of their application [18]. *S. pyogenes* usually resides in the upper respiratory tract, but during co-infection with IAV, transmission to the lower respiratory tract occurs and leads to the development of severe pneumonia [19]. Further severe diseases associated with *S. pyogenes* infections secondary to IAV are septicemia and necrotizing fasciitis [20].

The synergistic lethality of IAV and bacterial coinfection suggests a causative relationship between IAV infection and secondary bacterial pneumonia. Enhanced susceptibility to a secondary bacterial infection following IAV infection has been well recognized [21–23]. Adherence to host tissue is a critical initial step to establish bacterial infection and increased bacterial adherence in the context of IAV infection has been reported. IAV infection of human lung epithelial cells increased TGF-β activity and resulted in increased adherence of *S. pyogenes* [24]. In a murine infection model, IAV infection promoted invasive *S. pyogenes* infection by hemagglutinin (HA) expression on the surface of epithelial cells, which supported adherence and internalization of the bacteria [25]. IAV has been also shown to induce cyclophilin A expression in infected cells, thereby promoting integrin α5 expression and actin rearrangement, leading to increased bacterial infection [26]. Direct interaction of capsulated *S. pyogenes* with IAV particles has been demonstrated and might play a role in host tissue invasion [27]. Furthermore, the M protein and the fibronectin/tenascin binding protein PrtF.2 contribute to IAV superinfection [28, 29]. These examples demonstrate that IAV infection in many cases affects host-pathogen interaction, thereby promoting bacterial superinfection.

Although *S. pyogenes* is not the most common cause of bacterial superinfection in IAV epidemics, its mortality is surprisingly high. During the 2009 H1N1 IAV pandemic, 7 of 10 patients in California with 2009 H1N1 influenza and concurrent invasive group A streptococcal infection died, despite treatment with antibiotics and antiviral agents [30]. Collectively, despite the availability of influenza vaccines and numerous antibiotic treatment options for *S. pyogenes*, IAV-*S. pyogenes* superinfections continue to result in significant mortality [20]. However, despite extensive research on individual pathogens, host-directed therapeutic approaches targeting the common molecular pathways disrupted during IAV-*S. pyogenes* coinfections remain largely unexplored.

In this context, drug repurposing - the identification of new therapeutic uses for existing pharmaceutical compounds - represents a cost- and time-effective approach for discovering novel treatments [31]. By leveraging compounds with established safety profiles, pharmacokinetics, and bioavailability, drug repurposing significantly reduces the financial risks and development timelines associated with traditional drug discovery [32–34]. The advent of high-throughput gene expression databases has further enhanced this approach by enabling the development of algorithms that can predict therapeutic effects based on molecular signatures [35]. Therefore, we sought to identify novel host-directed therapeutic options for IAV-*S. pyogenes* coinfections by characterizing shared transcriptional signatures during individual pathogen infections and leveraging computational drug repurposing to identify compounds capable of reversing these host responses.

Accordingly, in this work we sought to identify novel host-directed therapeutic options for the *S. pyogenes*-IAV coinfection through computational drug repurposing. For this, we used a human pharyngeal epithelial cell line (Detroit 562) as model, performed single infection experiments with either IAV or two strains of *S. pyogenes* (M1 and M49), and then RNA-seq analysis was employed to uncover the host responses at the gene expression level. The focus on single infections is due to the known synergistic effects of Streptococcal species and IAV. Therefore, it is necessary to determine the effects of each pathogen individually. This knowledge can then be applied in future RNA-seq studies involving both pathogens. The identified differentially expressed genes (DEGs) were mostly associated with mitochondrial functions, signal transduction, and inflammatory responses, and then used as input for computational drug repurposing, in order to find compounds that could potentially reverse the molecular phenotype during these infections in the host cell. The predicted compounds included the antitumoral and antibacterial compound mitoxantrone, as well as inhibitors of cyclin-dependent kinases (AT7519 and flavopiridol) and Polo-like kinase 1 (BI2536).

## Results

We performed single infections of a human pharyngeal epithelial cell line (Detroit 562) using either Streptococcus pyogenes serotype M1 strain AP1, serotype M49 strain 591, or influenza A virus (IAV). Total RNA was isolated from these infections for dual RNA-seq analysis. This experimental design allowed us to identify common host responses to each pathogen while simultaneously monitoring pathogen-specific transcription profiles during infection. S. pyogenes transcription was analyzed to compare infection-related gene expression between the two bacterial strains, with the aim of identifying potential bacterial pathways as targets for drug repurposing, while IAV transcripts were used as indicators of successful viral infection.

### Mono-infection of detroit 562 with *S. pyogenes* or IAV

Detroit 562 cells were infected with either *S. pyogenes* M1 AP1 (M1 AP1) or serotype M49 strain 591 (M49 591) at a multiplicity of infection (MOI) of 100. IAV infection was performed with 500 plaque forming units (PFU) of IAV per well. Total RNA was isolated for RNA sequencing and infection parameters were determined to control for the experimental conditions.

**Fig. 1:**
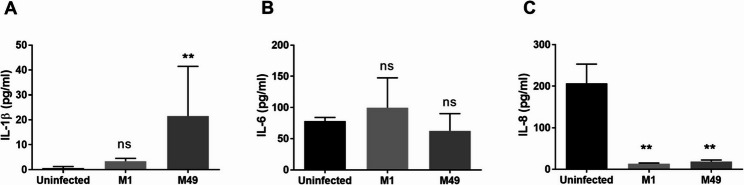
*S. pyogenes* infection affects pro-inflammatory cytokine concentration in cell culture supernatants. Supernatant of cells infected with a MOI 100 of S. pyogenes were collected 2 hpi. Concentration of IL-1β (**A**), IL-6 (**B**) and IL-8 (**C**) was determined by ELISA in duplicates. Each experiment was repeated three times. Data are presented as mean values with error bars representing the standard deviation. Asterisks indicate significant differences compared to the non-infected control as determined by One-way ANOVA, Dunn’s multiple comparison test, with p-values <0.01 (**)

Bacterial infection of Detroit 562 was monitored by assessing bacterial adherence, host cytokine response, and the extent of host cell death. 25‑35% of *S. pyogenes* cells adhered to Detroit 562 two hours post infection (hpi). IFN‑γ, IL-10, and TNF-α were not present in detectable concentrations in the supernatants, regardless of the condition tested. The concentration of IL-1β in the cell culture supernatant was not affected 2 hpi with *S. pyogenes* M1 AP1 compared to non-infected control samples (Fig. 1A) but increased 34-fold 2 hpi with *S. pyogenes* M49 591. Secretion of IL-6 was not affected by infection with *S. pyogenes* (Fig. 1B). In contrast, the concentration of IL-8 in the supernatant dramatically decreased following infection compared to the non-infected control (Fig. 1C). IL-8 concentration decreased following infection. This reduction is consistent with bacterial cell envelope proteinase SpyCEP activity, indicating successful streptococcal infection [36].

**Fig. 2:**
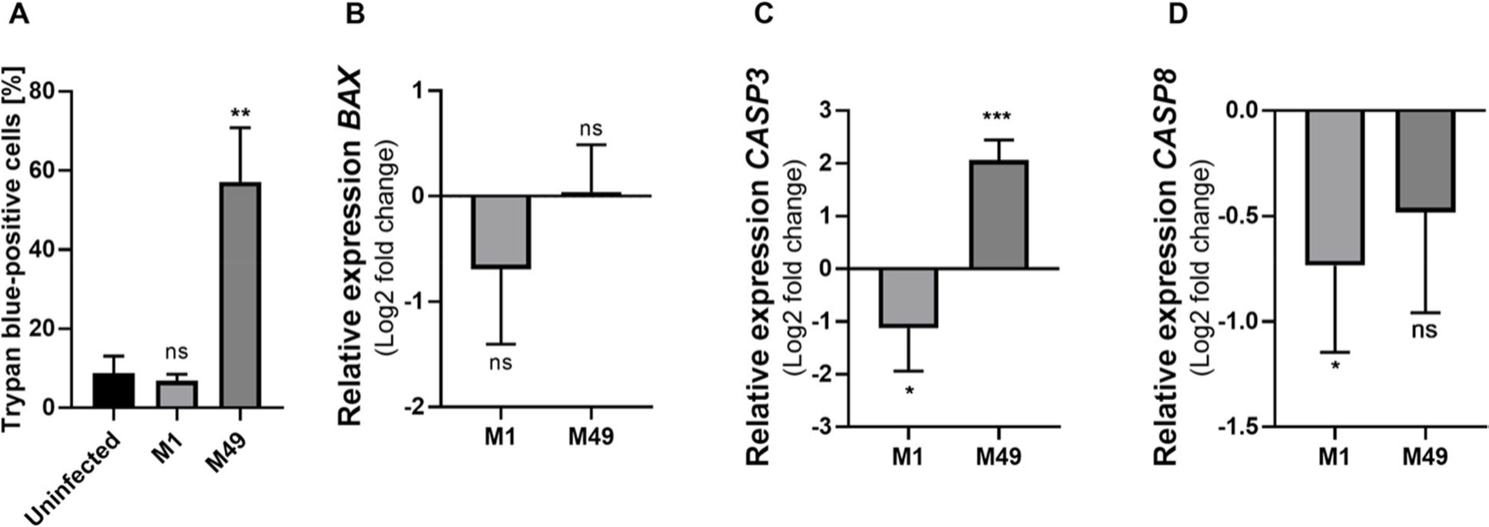
*S. pyogenes* infection causes cell death and affects the expression of cell death markers. **A** Trypan blue staining of Detroit 562 cells was performed 2 hpi with S. pyogenes M1 AP1 or serotype M49 strain 591. Each experiment was repeated four times. Error bars represent the standard deviation of the mean value. Significant differences compared to the non-infected control were determined by One-way ANOVA, Dunn’s multiple comparison test. p-value <0.01 (**). **B-D** Relative transcript abundance of BAX (**B**), CASP3 (**C**), and CASP8 (**D**) in GAS-infected Detroit 562 cells was determined by RT-qPCR. Each experiment was repeated four times. Error bars represent the standard deviation of the mean value. Significant differences compared to the non-infected control were determined by One-way ANOVA, Dunnett’s multiple comparison test. p-value <0.001 (***) and p-value <0.05(*)

Trypan blue dye exclusion was performed to monitor host cell death. Infection with *S. pyogenes* M1 AP1 did not cause host cell death (Fig. 2A). In contrast, 2 hpi with *S. pyogenes* M49 591, approximately 60% Detroit 562 cells were trypan blue-positive, indicating considerable cell death under these conditions. Relative transcript abundance of the cell death-related markers *BAX*, *CASP3*, and *CASP8* was determined from total RNA employing reverse transcription followed by qPCR (Fig. 2B-C). Infection with *S. pyogenes* did not influence mRNA levels of *BAX* (Fig. 2B) in Detroit 562. Relative transcript abundance of *CASP3* decreased following infection with *S. pyogenes* serotype M1 strain AP1, whereas infection with *S. pyogenes* M49 591 increased relative expression of *CASP3* (Fig. 2C). Expression of *CASP8* was slightly reduced following infection with *S. pyogenes* M1 AP1 (Fig. 2D), whereas infection with *S. pyogenes* M49 591 did not reduce *CASP8* expression significantly. Overall, infection of Detroit 562 with 100 MOI of *S. pyogenes* led to pathogen-specific host cell responses. Specifically, infection with *S. pyogenes* M49 591 caused dramatically increased secretion of IL-1β and excessive cell death in correlation with increased relative expression of the cell death marker *CASP3.* In contrast, the effects of Detroit 562 infection with *S. pyogenes* M1 AP1 were comparably mild. Neither elevated secretion of pro-inflammatory cytokines nor increased expression of cell death markers were observed. Accordingly, no cell death occurred following infection with this strain.

**Fig. 3:**
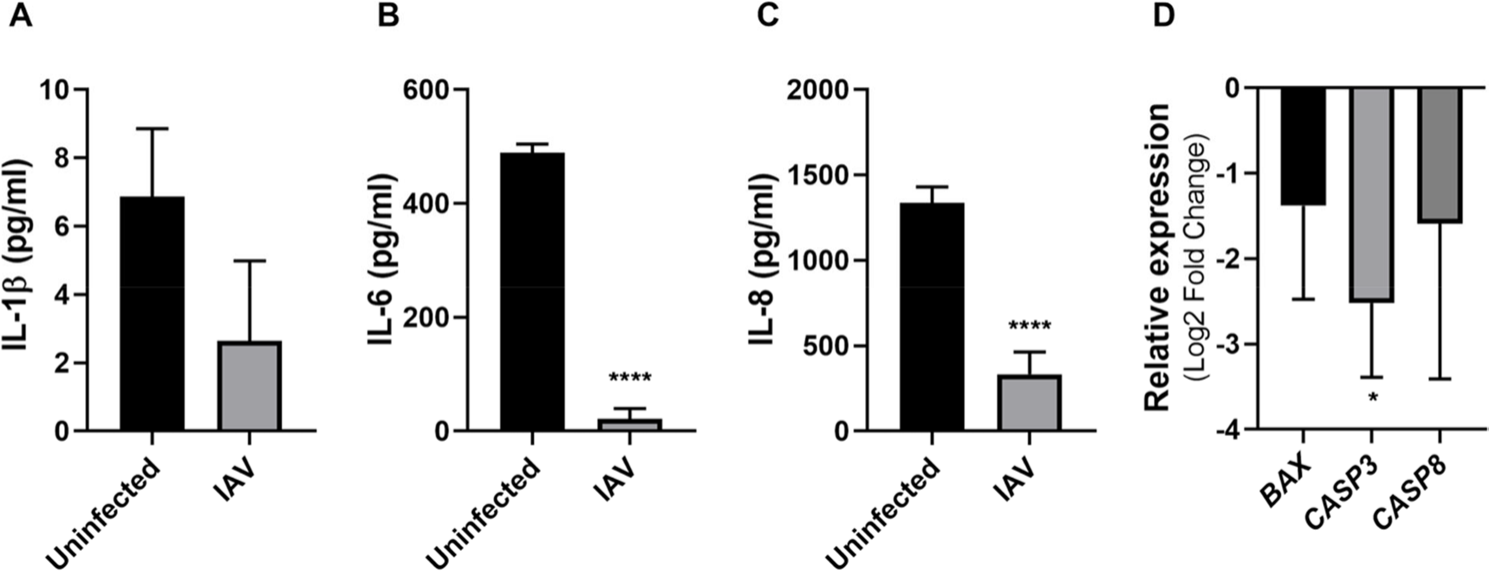
Effects of IAV infection on Detroit 562 cells. **A-C** Cells were infected with 500 PFU of IAV for 48 h. Concentration of IL-1β (A), IL-6 (B) and IL-8 (C) was determined by ELISA. Each experiment was repeated three times. Error bars represent the standard deviation of the mean value. Significant differences compared to the non-infected control were determined by Unpaired t test, p-value <0.0001 (****). **D** Relative transcript abundance of *BAX*, *CASP3*, and *CASP8* in IAV-infected Detroit 562 cells was determined via RT-qPCR. Each experiment was repeated four times. Error bars represent the standard deviation of the mean value. Significant differences compared to the non-infected control were determined by One-way ANOVA, Dunnett’s multiple comparison test, p-value <0.05(*)

Host cell response to IAV infection was assessed by measuring cytokine concentrations in culture supernatants. Infection with 500 PFU IAV resulted in dramatic decreases in pro-inflammatory cytokine secretion, with IL-1β, IL-6, and IL-8 levels all falling significantly below control levels (Fig. 3A). Despite these inflammatory changes, trypan blue exclusion assays revealed no increase in cell death at 48 hpi compared to non-infected controls (data not shown). Consistent with this observation, the relative expression of apoptosis-related markers BAX, CASP3, and CASP8 was downregulated following IAV infection (Fig. 3B-D). These findings indicate that IAV infection suppresses both pro-inflammatory cytokine production and apoptotic signaling pathways in Detroit 562 cells without causing significant cell death during the 48-hour experimental period.

### Mapping depth of the dual RNA-Seq of detroit 562 mono-infections

Infection of Detroit 562 was performed under the conditions described above. 2 hpi with *S. pyogenes* and 48 hpi with IAV, respectively, cells were collected and total RNA was isolated. Non-infected Detroit 562 cells harvested at the corresponding time points served as control. Each experiment was performed in three independent biological replicates. 1 µg total RNA per sample was used for the synthesis of rRNA-depleted cDNA libraries. To prevent removal of bacterial RNA, polyA+ selection was avoided. The libraries were sequenced on a NextSeq500 platform (Illumina) using a 75 bp single reads sequencing protocol. The sequencing runs delivered 32 million raw reads on average. After trimming, ~ 99.74% were retained from each sample. Trimmed reads were mapped to their respective reference genomes. 92–98% of the total raw reads were attributed to Detroit 562 (average coverage human exome of 77.6). Total raw reads originating from the pathogens were 0.27% of *S. pyogenes* serotype M1 strain AP1 (average coverage of 3.6), 4.02% of *S. pyogenes* serotype M49 strain 591 (average coverage of 55.6), and 0.06% of IAV (average coverage of 110.3), respectively (Supplementary Table 1). Gene quantification for infection libraries revealed an average Detroit 562 gene count of 26% of the total raw reads. Pathogen gene counts were determined to be 0.02% of total raw reads for *S. pyogenes* serotype M1 strain AP1, 0.04% for *S. pyogenes* serotype M49 strain 591, and 0.01% for IAV (Supplementary Table 1).

Afterwards, to confirm that the sample size and sequencing depth were sufficient to display significant differences between the infection groups, a post-hoc rarefaction curve and power optimization analysis was conducted with the Scotty tool, from the obtained RNA-seq reads mapped to host or pathogen genes [37]. Rarefaction curves were used to compare the abundance of genes in samples with different sequencing depth and to determine whether the amount of sequenced reads is sufficient to assess diversity at the gene level [38]. In general, rarefaction curves display an early exponential phase, in which a large number of genes remains to be discovered, and slowly approach a plateau phase indicating that more sampling will not lead to the discovery of more genes. The presence of a plateau phase suggests that the count table is a good representation of the diversity in the sample [39]. Here, the number of genes detected with 10 or more reads was plotted as a function of read depth. Our analysis showed that the rarefaction curves of the Detroit 562 reads reached the plateau phase (Supplementary Fig. 1). In contrast, rarefaction curves of *S. pyogenes* reads did not saturate, suggesting that the number of sequenced reads is too low to represent transcript diversity (as previously suggested by the mapping coverage). Therefore, bacterial expression data were excluded from further analyses. In addition, rarefaction curves were not generated for IAV because the analysis is not suitable for the low numbers of mapped reads.

Similarly to the rarefaction curves, the Poisson Noise estimates the ability of the count table to represent the diversity of the genes present in the sample. Our analysis determined values between 77.94% and 86.41% for the human gene count between the infection conditions and the control samples. Thus, 14%-22% of the lowly expressed genes from the human host are under-represented (Supplementary Fig. 1B). The Power Optimization calculates the probability of the count table to identify all DEGs between two groups. The probability to identify all host DEGs between IAV infected Detroit 562 and the uninfected control was 61.04%. For samples from bacterial infection conditions, the probability was 70.78% (M1) or 72.87% (M49), respectively. In summary, reads mapped to pathogen genes were detected in all the infection libraries, but as the rarefaction curves showed that these do not satisfactorily represent the gene diversity of the pathogens in the samples, only mapped reads for the host genes were used for further analyses.

Afterwards, and given the different sequencing coverages and in order to avoid spurious alignments and obtaining comparable numbers of differentially expressed genes (DEGs), data was filtered for infection-specific minimum thresholds of 400 total mapped reads for M1 infections, 1,800 reads for M49 infections, or 600 reads for IAV infections for each of the host genes, when counting both the control and infected RNA-Seq replicates combined. Application of these filtering thresholds retained 10,368 genes for differential expression analysis in M1 infections, 6,068 genes in M49 infections, and 9,388 genes in IAV infections. These retained gene sets provided sufficient statistical power for reliable detection of differentially expressed genes, as confirmed by post-hoc power analysis using Scotty at 2-fold change (*p* < 0.05).

### Transcriptional dynamics in the host upon infection with *S. pyogenes* M1 AP1 and M49 591

We identified 84 DEGs for the M1 infection sequencing samples (55 upregulated and 29 downregulated); and 1,650 DEGs for the M49 infection (841 upregulated and 809 downregulated; Supplementary File 1), and then we filtered these results by log2- fold changes (LFC; |LFC| > 0.25) for the M1 infection RNA-seq data, to focus only on dramatic changes in expression. The gene annotations for the identified DEGs for the M1 infection at the biological process category (GO: BP, Supplementary Fig. 2A) can be summarized as DNA replication-dependent chromatin assembly (GO:0006335) for the downregulated genes, and into mitochondrial ATP synthesis function terms for the upregulated genes. Similarly, the molecular function annotations (GO: MF; Supplementary Fig. 2B) for this infection were summarized into NADH and NAD(P)H enzyme activity on the transmembrane transportation for the upregulated genes, and GO:0017017 “MAP kinase tyrosine/serine/threonine phosphatase activity” for the downregulated DEGs. The top 25 upregulated genes in the M1 infection (Supplementary Table 2) are mostly associated to apoptosis, immune response, vascular functions, transcriptional regulation and signaling, protein transport and mitochondrial functions; while the top 25 downregulated genes (Supplementary Table 3) are associated to signaling, transcription regulation, ion transport, and chromosome organization.

Similarly, we filtered the DEGs for the M49 infection data for LFCs (|LFC| > 1.5), and in this manner we obtained 72 upregulated and 33 downregulated genes. The upregulated DEGs in this context are linked to mitochondrial ATP production and positive regulation of transcription from the RNA polymerase II due to stress response for the GO: BP annotation; and the regulation of the NADH and NAD(P)H activity for the GO: MF annotation (Supplementary Fig. 2C-D). The top 25 upregulated genes in the M49 infection (Supplementary Table 4) are associated to the regulation of transcription, cell adhesion, signaling, DNA damage response, immune response, apoptosis, differentiation, and mitochondrial genes; while the top downregulated genes (Supplementary Table 5) are related to the extracellular matrix organization, ion transport, transcriptional regulation, differentiation, signaling, cell migration and proliferation.

Later, to find the processes common to both the M1- and M49- infected cells, we first identified the shared DEGs for both infections (Supplementary Table 6). These genes are related to signal transduction, DNA damage response, immune response, regulation of transcription, protein transport, migration, xenobiotic metabolism, ribosome biogenesis, zinc ion transport, and mitochondrial genes and pseudogenes. In all cases, these genes were found up- or downregulated in both infection models, except for the endothelin 2 gene END2, which was upregulated in M1 and downregulated in M49 infections (Supplementary Table 6). The gene ontology annotations of these 24 DEGs (Supplementary Table 7) can be summarized in the following terms: electron transport system (GO:0015986, GO:0015990, GO:0022904) and mitochondrial ATP production (GO:0032981). The MegaGO analysis showed a high level of functional relatedness between the gene ontology annotations of the DEGs from the M1- and M49- infected cells (from 0.7 to 0.92, Supplementary Table 8) [40].

Later, we identified 57 and 63 DEGs specific to M1- and M49- infected cells, respectively. The M1-specific DEGs were linked to the following ontologies: negative regulation of transcription (NFIL3, ZNF217, PRDM1, DLX2, CITED2, HDAC9, ID2, BHLHE40, SMAD7, KLF4), negative regulation of DNA- binding transcription factor activity (BHLHE40, SMAD7, KLF4, ID2, HES1, TRIB1), astrocyte differentiation (ID2, HES1, CLCF1), and negative regulation of muscle cell migration (TRIB1, SERPINE1, IGFBP3); while the M49- specific are associated to the response to endoplasmic reticulum stress (JUN, HERPUD1, CHAC1, PPP1R15A, ATF3, DDIT3), positive regulation from RNA polymerase II promoter in response to stress (ATF3, DDIT3, SESN2, VEGFA), extracellular matrix disassembly (MMP1, MMP7, MMP10, CTSV, ADAM8), and cellular response to cytokine stimulus (MMP1, VEGFA, EGR1, ZFP36, CEBPD, FN1, PTPRZ1, DUSP1, SOCS3, ARHGEF2, FOS).

### Transcriptional dynamics in the host upon infection with IAV

Here we found 3,856 DEGs (2,004 upregulated and 1,852 downregulated), upon a cutoff of 600 total reads per gene. These results were further filtered for expression fold changes (| LFC | > 2.5); and as a result we obtained 116 upregulated and 46 downregulated genes for the IAV infection, which were used in further analyses. The GO: BP of the upregulated DEGs (Supplementary Fig. 2E) can be described with the following terms: peptide cross-linking (GO:0018149), intermediate filament-based process and organization (GO:0045103, GO:0045109, GO:0045104); while the GO: BP of the downregulated DEGs described processes related to the regulation of nitric-oxide synthase biosynthesis (GO:0051770 and GO:0006809). The GO: MF annotations of the upregulated DEGs correspond to the “structural constituent of skin epidermis” ontology (GO:0031424, Supplementary Fig. 2F). The GO: MF of the downregulated DEGs could not be further summarized. Besides, we did not find functional relatedness between the GO annotations from the M1 and IAV DEGs and the M49 versus IAV annotations, according to the MegaGO analysis [40] (Supplementary Table 8).

In this regard, the top 25 upregulated genes (Supplementary Table 9) are associated to apoptosis, proliferation, ion transport, differentiation, immunity, signaling or signal transduction, protein transport, xenobiotic metabolism and migration; while the top 25 downregulated genes (Supplementary Table 10) are related to the immune response, signal transduction, differentiation, transcriptional regulation, proliferation, migration, cell adhesion, apoptosis, ion transport and nucleosome.

### Transcriptional changes in the host common to *S. pyogenes* and IAV infections

When comparing all DEGs sets we found four genes expressed in all M1- M49- and IAV- infected cells: the arrestin domain containing 3 (ARRDC3), FosB proto-oncogene, AP-1 transcription factor subunit (FOSB), URB2 ribosome biogenesis homolog (URB2), and DNA damage inducible transcript 4 (DDIT4). The STRINGdb network analysis of these four genes revealed that ARRDC3 is associated with FOSB through interactions with the ubiquitin ligase NEDD4 and the signal transduction proteins SMAD3 and FOS respectively, while DDIT4 is linked to FOSB through interactions with the transcriptional repressor TXNIP, the histone acetyltransferase EP300, and the above mentioned SMAD3 and FOS (Supplementary Fig. 3). Furthermore, a search on the Harmonizome database [41] showed that ARRDC3, DDIT4 and URB2 are targets for the FOS transcription factor gene family, to which FOSB belongs, as proven experimentally with chromatin immunoprecipitation followed by sequencing (ChIP-seq) experiments, and reinforces our results that closely associate these four genes in this transcriptional response against infection.

Later, when comparing the DEGs from all groups before applying the LFC filters, we found 28 DEGs common to all three infections: 18 upregulated genes in all 3 groups (except for FOSB, as shown above), including the aforementioned FOSB and DDIT4, as well as transcription factors (KLF6, NFKBIZ), the nucleotidyltransferase TENT5A and mitochondrial genes (MT-ND4, MT-ND4L, MT-CYB, MT-CO1, MT-CO3, MT-ND2); and 10 downregulated, entailing genes associated with the epigenetic control (HDAC9, H4C2, H4C13, H3C12), besides of two paralogs of the sodium transporter SLC20A1 gene (Supplementary Table 11; Supplementary Fig. 4). Gene ontology analysis of these common DEGs revealed distinct functional signatures for upregulated and downregulated genes (Supplementary Table 12). According to g: Profiler, the upregulated genes showed enrichment for “response to hypoxia” and “ATP metabolic process” as driver terms, consistent with metabolic reprogramming during viral infection. Conversely, the downregulated genes were enriched for “structural constituent of chromatin” and “nucleosome assembly” driver terms, reflecting chromatin remodeling during infection.

### Transcriptional changes in the host specific to either *S. pyogenes* and IAV infections

As for the infection- specific genes, we found 17, 686 and 2,887 DEGs specific for M1- M49- and IAV- infected cells respectively, when comparing sets of DEGs before applying the LFC filters. The top significant annotations for the M49 infection DEGs are endodermal cell differentiation (GO: BP), metabolic pathways of fibroblasts and IL-2 signaling pathway. The DEGs specific for the IAV- infected cells are linked to the structural constituent of nuclear pore, exonuclease activity, snoRNA binding ontologies, and the DNA replication and DNA mismatch repair pathways. The 17 genes specific for the M1 infection include the small nucleolar RNA (snoRNA) SNORA74A, and 16 protein coding genes. The GO annotation showed an overall highlight on structural binding of both intracellular and extracellular matrix with the only GO: MF term with GO:0005488 (binding); while the identified GO: BP term is related to the negative regulation of endothelial cell apoptotic process (GO:2000352); and the GO: CC annotation with terms such as collagen-containing extracellular matrix (GO:0110165) and intracellular membrane-bounded organelle (GO:0043231).

### Computational prediction of candidate compounds to reverse the expression profile in the host

**Fig. 4:**
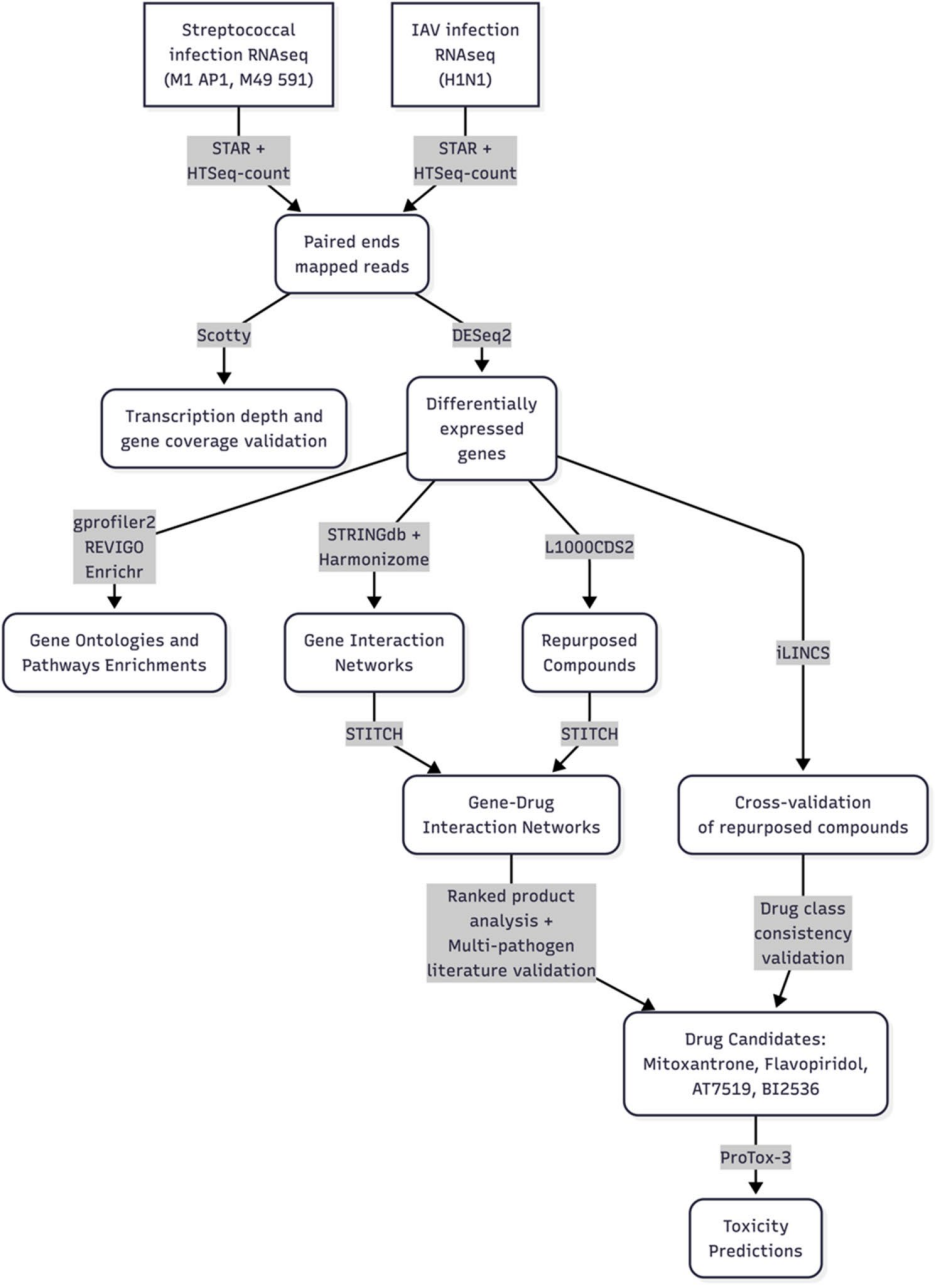
Bioinformatics workflow for the transcriptomic analysis and computational drug repositioning. The main components of the workflow are as follows. Following RNA-seq experiments from three infection models (S. pyogenes M1, S. pyogenes M49, and IAV), Illumina reads were quality-controlled and mapped against the human reference genome (GRCh38) using STAR aligner. Read counts per gene were quantified with HTSeq-count and used for differential gene expression analysis with DESeq2. Transcription depth and gene coverage were assessed using Scotty to validate sequencing adequacy. Differentially expressed genes (DEGs) were functionally annotated through gene ontology and pathway enrichment analysis using gprofiler2, with results summarized via REVIGO and extracted with Enrichr. Gene interaction networks were constructed using STRINGdb and Harmonizome databases. For drug repositioning, DEGs were queried against the L1000CDS2 server to identify compounds capable of reversing infection-induced transcriptomic signatures. Finally, top-ranked drug candidates and DEGs were integrated into comprehensive gene-drug interaction networks using the STITCH database to elucidate potential therapeutic mechanisms

Since key cellular processes related to the inflammation were significantly enriched in the common differentially expressed genes from the streptococcal and viral infections (Supplementary Table 12; Supplementary Fig. 2), we hypothesized that reversing this expression state might enhance the immune response against these infections. Thus, we queried the gene expression signatures using the L1000CDS2 tool to identify small molecules that exhibit a reverse expression pattern compared to those observed during the infection processes. These molecules could potentially be used to enhance the human immune response against these infections (Supplementary File 2). The top five ranked compounds are listed for each infection in Supplementary Table 13. Then, to find potential drugs that are effective in different infections, we calculated the ranked product by multiplying the ranks from each compound for the pairs of infections and the three infections simultaneously [42]. In this manner we found seven compounds predicted for the M1 and M49- infected cells, and two for the M49 and IAV- infected cells (Table 1). Afterwards, to select further the compounds predicted with L1000CDS2, we analyzed the drug-gene interactions between these candidate compounds and the sets of DEGs. For this, we combined the predicted compounds (Table 1) together with the DEGs for each of the three infection groups and used these as input for the STITCH server, from which we obtained scores of drug-gene interactions (Table 2). The whole analysis workflow is depicted on Fig. 4. Here we notice that STITCH found additional compounds, some of which interacting in more than one infection simultaneously: alvocidib in M1 and IAV, CGP-60,474 in M49 and IAV, and A-443,654 in M1 and IAV infections.

**Table 1:** Drugs predicted as reversing in more than a single infection

Broad Id	Perturbation	Rank Product	Infection
BRD-K87909389	alvocidib (flavopiridol)	7	M1 and M49
BRD-K79090631	CGP-60,474	24	M1 and M49
BRD-A60245366	AS-601,245	135	M1 and M49
BRD-K13390322	AT-7519 (AT7519)	192	M1 and M49
BRD-K88573743	A-443,654	207	M1 and M49
BRD-K21680192	mitoxantrone	248	M1 and M49
BRD-K19220233	JNK-9 L	462	M1 and M49
BRD-K96799727	Pifithrin-mu (2- phenyl ethane sulfonamide)	168	M49 and IAV
BRD-K64890080	BI-2536 (BI2536)	690	M49 and IAV

Rank products were calculated by multiplying the ranks for each compound across infection pairs, e.g., for alvocidib, 7 represents the product of the ranks obtained from the L1000CDS2 predictions for the M1 and M49 DEGs. Each drug is shown with their corresponding Broad Database id (“Broad id”), their rank product score, and the infections from which these rank products were calculated

**Table 2:** Predicted gene – drug interactions for each infection group

Accession	Gene	Compound	Score	Infection
ENSP00000269305	TP53	alvocidib (flavopiridol)	0.88	M1
ENSP00000269305	TP53	Pifithrin-mu (2-Phenylethynesulfonamide)	0.85	M1
ENSP00000269305	TP53	A-443,654	0.8	M1
ENSP00000389338	MAPK9	JNK inhibitor V	0.73	M49
ENSP00000353483	MAPK8	JNK inhibitor V	0.71	M49
ENSP00000447803	DDIT3	CGP-60,474	0.45	M49
ENSP00000396308	DHFR	methotrexate	1	IAV
ENSP00000266970	CDK2	AT-7519 (AT7519)	0.97	IAV
ENSP00000266970	CDK2	alvocidib (flavopiridol)	0.96	IAV
ENSP00000266970	CDK2	CGP-60,474	0.62	IAV
ENSP00000259206	IL1RN	methotrexate	0.48	IAV
ENSP00000266970	CDK2	A-443,654	0.439	IAV

Genes are listed with their corresponding ENSEMBL peptide ids and gene symbols (“Accession” and “Gene” respectively), together with the compound their interact (“Compound”), and their interaction score (“Score”), for each infection group. These interactions were predicted using the STITCH server

### Cross-validation of drug predictions using connectivity map signatures

To validate our L1000CDS2-based drug predictions, we performed cross-platform analysis using the iLINCS server with original Connectivity Map signatures [43]. This analysis revealed both exact compound matches and drug class consistency across platforms. Among our four primary candidates, mitoxantrone appeared in the iLINCS predictions for M1 infections with a z-score of 7.05 (ranked 113 of 382 compounds), directly confirming this prediction across independent databases. More broadly, we observed strong drug class consistency validating our computational approach. PLK1 inhibitors appeared across all three infections in the iLINCS results, with wortmannin ranking particularly well in IAV infections (rank 47 of 223 compounds), supporting our BI2536 prediction which also targets PLK1. The cyclin-dependent kinase inhibitor staurosporine appeared in M1 infections (rank 239 of 382), supporting our predictions of flavopiridol and AT7519. This drug class consistency demonstrates that different connectivity mapping platforms converge on the same predicted therapeutic mechanisms even when they do not identify identical compounds. The relatively low rate of exact compound matches between platforms is consistent with published concordance rates between Connectivity Map and L1000 databases, which typically show approximately 17% overlap [44]. This reflects differences in compound libraries (519 compounds in CMap versus over 140,000 signatures in L1000), experimental platforms, and signature generation methods rather than invalidating either approach. The biological consistency in identifying kinase signaling pathways as therapeutic targets across both platforms strongly supports the validity of our drug repositioning strategy. Complete iLINCS results are provided in Supplementary File 4.

### Mechanistic analysis of candidate drug-gene interactions

**Fig. 5:**
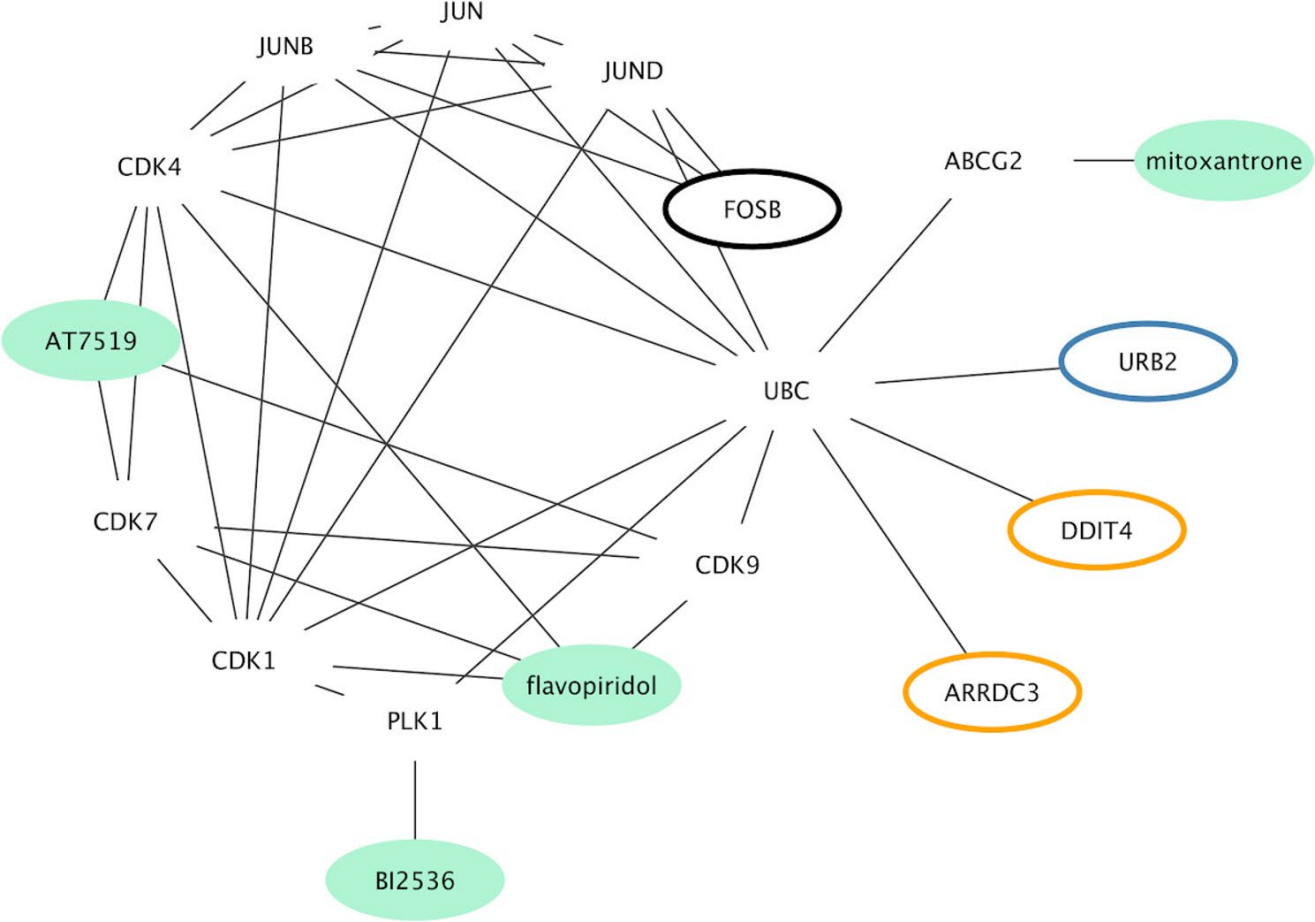
Candidate drug – gene interaction network for the S.pyogenes and IAV coinfections. Network of genes found as DEGs in all infections (ARRDC3, DDIT4, FOSB and URB2), combined with (**a**) compounds predicted to counter these infections based on L1000CDS2 (Supplementary Table 13), and (**b**) other genes predicted as interacting by STITCH with high confidence (edge confidence score > 0.7, indicating significant interactions; [45]; accessed 2023-02-01; parameters: species Homo sapiens, discarded “Predictions” from active interaction sources). Node border colors indicate upregulation (orange) or downregulation (blue) in all three infections, or infection-specific up- or downregulation (thick black border). Genes not found as DEGs in all infections but predicted as interacting are shown as nodes without borders, and compounds are depicted as green nodes

In order to study the candidate mechanism of action of the predicted drugs, we took the genes that appear in all sets of DEGs (ARRDC3, FOSB, URB2, DDIT4) and combined them with the drugs predicted by L1000CDS2 for DEGs appearing in all infections (Table 3), as input for a query on the STITCH database. We found that these four DEGs interact with cyclin-dependent kinases (CDK4, CDK9, CDK7, CDK1), either through interactions with proteins from the JUN family (FOSB) or via interactions with the ubiquitin C (ARRDC3, DDIT4, URB2); and through these CDK kinases they are predicted to be modulated by mitoxantrone, BI 2536, flavopiridol, and AT7519 (Fig. 5). In particular, flavopiridol and AT7519 are the compounds that display the largest number of interactions (all of them through CDKs) and with reliable scores (edge confidence > 0.7). Both are CDK inhibitors and through this mechanism are predicted to induce apoptosis in infected cells.

**Table 3:** Predicted gene – drug interactions for the candidate network of the three infections

Accession	Gene	Compound	Score
ENSP00000237612	ABCG2	mitoxantrone	0.998
ENSP00000300093	PLK1	BI-2536 (BI2536)	0.998
ENSP00000257904	CDK4	alvocidib (flavopiridol)	0.997
ENSP00000362361	CDK9	alvocidib (flavopiridol)	0.997
ENSP00000256443	CDK7	alvocidib (flavopiridol)	0.996
ENSP00000378699	CDK1	alvocidib (flavopiridol)	0.99
ENSP00000256443	CDK7	AT-7519 (AT7519)	0.914
ENSP00000362361	CDK9	AT-7519 (AT7519)	0.908
ENSP00000257904	CDK4	AT-7519 (AT7519)	0.881

Listed here are DEGs appearing in all three infections (with their respective ENSEMBL id and symbols), together with the repurposing drugs predicted by L1000CDS2, and their corresponding interaction score, as calculated by STITCH

### Computational toxicity assessment of candidate compounds

To assess potential safety concerns with our candidate compounds, we performed computational toxicity predictions using the ProTox-3 server [46]. Acute toxicity predictions revealed that mitoxantrone showed a predicted LD50 of 46 mg/kg (toxicity class 4, harmful), flavopiridol 800 mg/kg (class 4, harmful), AT7519 420 mg/kg (class 4, harmful), and BI2536 1000 mg/kg (class 4, moderately toxic). All four compounds showed inactive predictions for hepatotoxicity and carcinogenicity, suggesting lower risk for these serious adverse effects. Regarding blood-brain barrier penetration, flavopiridol, AT7519, and BI2536 showed predicted BBB penetration, whereas mitoxantrone lacked this activity. Additional organ-specific toxicity predictions showed inactive results for most endpoints across all compounds. Complete toxicity prediction data including all assessed endpoints are provided in Supplementary File 3.

## Discussion

IAV infection in combination with a secondary bacterial infection often leads to a severe clinical outcome with a higher mortality rate when compared to single event of infection. *S. pyogenes* is one of the typical bacterial pathogens found in coinfections with IAV. Classical treatment with antiviral agents and antibiotics is often insufficient to prevent severe complications. In this work, we sought to identify common host-specific pathways that are differentially activated in IAV and streptococcal infections by transcriptomic analysis that have the potential to increase the mortality rate in a IAV-*Streptococcus* comorbidity in humans. The resulting data were used in order to predict novel compounds by computational drug repurposing that support specifically the immune response to IAV-*S. pyogenes* co-infection.

### Transcriptional changes in the host during infections with *S. pyogenes* M1 and M49

The effects of *S. pyogenes* infections on the gene expression in host cells have been extensively studied. For example, epithelial cell lines such as Detroit 562 (pharynx), HEp-2 (cervix), HaCaT (keratinocytes), A549 (lung) and TEpi (tonsil) express and/or secrete a vast collection of pro- inflammatory mediators, including chemokines, cytokines and growth factors [47]. Similar results were obtained with keratinocytes isolated from biopsies, where streptococcal superantigens elicited the production of the chemokines IL-8, IL-33 and MIP-3 [48]. In our case, both streptococcal infections showed an upregulation of mitochondrial genes related to ATP production. Moreover, the M49 infection also affect the regulation of transcription from the RNA polymerase II promoter. The similarity in DEG annotations in both M1- and M49- infected cells was later confirmed when we observed high scores of functional relatedness on all three GO categories with a 24 shared set of DEGs among the streptococcal infections. In this respect, NFKBIZ is one of the NF-kB inhibitors, whose degradation leads to the NF-kB translocation to the nucleus and the activation of immune responses mediated by this protein [49], while the upregulation of CYP1A1 has been observed in colorectal cell lines exposed to *S. gallolyticus* and other *S. bovis* group strains [50], and oral keratinocyte cell lines infected with *S. mitis*, but not with *S. gordonii* or *S. mutans* [51], suggesting that the activation of this cytochrome in host cells upon streptococcal infections is species- specific. Moreover and similarly to our results, the expression of FOS, DDIT4 and several DUSP phosphatases was highly upregulated upon *S. pneumoniae* infection of A549 cells [52], which indicates a common pro- inflammatory response against *S. pyogenes* and *S. pneumoniae* infections.

Furthermore, when analyzing the infection-specific DEGs, we identified two sets of negative regulators specific for the M1 infection, namely negative regulators of transcription and genes linked to the negative regulation of DNA-binding transcription factor activity; while DEGs specific for the M49- infected cells included genes linked to stress response, extracellular matrix disassembly, and response to cytokine stimulus. Noticeably, from the DEGs specific for the M1 infection, two have been already studied in similar contexts: The angiopoietin- like 4 (ANGPTL4) and the SERPINE1 genes. ANGPTL4, a gene associated with angiogenesis, has been found upregulated during influenza pneumonia and LPS- induced acute lung injury [24], and is regulated by the PPAR-gamma nuclear receptor and transcription factor, which in turn works as an immunomodulator by inhibiting NF-kB [53]; while SERPINE1 is a IFN- stimulated gene (ISG) encoding a serine protease inhibitor whose synthesis is stimulated in the nasal mucus in presence of *S. epidermidis* and plays a role in the restriction of the spread of IAV into the lungs [54, 55].

### Transcriptional changes in the host during the IAV infection

IAV infects several cell types, with epithelial cells from the upper respiratory tract as their main target, causing considerable cell death in these cells and consequently the destruction of the epithelial barrier. These high levels of cell death elicit inflammatory responses, with infected cells producing an array of chemokines and cytokines, and these pro-inflammatory responses cause even more damage to the tissues, allowing bacteria to spread into the host and causing a secondary infection. Therefore IAV infections increase the susceptibility to secondary bacterial infections (Reviewed in [56]). This is in agreement with previous reports that show the upregulation of cell death processes upon IAV infection [57, 58].

Previously, it has been observed that the influenza infection upregulates the expression of genes linked to the defense against viruses, in particular interferons (IFNs), interferon- stimulated genes (ISGs) and its associated pathways [57, 59, 60], and similarly for chemokine- pathway associated genes, such as CXCL-10, CXCL-9, CCL-7 and CXCL-2 in H5N8 and H5N1- infected mice [61], and cytokines and chemokines IL-6, IL-8, CCL-2, CXCL-1, CCL-3 and CXCL-3 in H1N1- infected lung epithelial (A549) cells [62]. However, specificity and variation in the host expression against different IAV strains has been previously observed, where H3N2 elicited the expression of IFNs and ISGs, while their expression was not significantly changed upon H1N1 infection [61], and these expression differences can be observed even within substrains of the same influenza virus type [60].

Moreover, we found that certain genes associated to the immune response, namely the chemokine ligand 2 CCL-2 and the leukemia inhibitory factor (LIF; a member of the IL-6 cytokine family), two genes from pathways linked to inflammation such as LBH (LBH regulator of WNT signaling pathway) and TLR4 (Toll-like receptor 4), as well as two genes that interact with integrins (CCN1, CCN2) were strongly downregulated. CCL-2 plays a central role in the recruitment of innate immune cells to the lung, as the interaction with its ligand, the CC chemokine receptor 2 (CCR2), is key in the monocyte migration during inflammation [63]. Previous reports show an increase of CCL2 expression upon influenza infection [57, 63, 64], while at the same time CCL-2 levels were significantly reduced in IAV-infected mice [65] and CCR2- deficient knockout mice were better protected from secondary bacterial infections [63], so the impact of CCL-2 in the outcome of secondary bacterial infections post-influenza depends on context [63].

In turn, TLR4 is a member of the Toll-like receptor family, which consists of transmembrane receptors involved in sensing pathogen molecules, with TLR4 involved in the recruitment of the NF-kB transcription factor, required for the induction of most inflammatory genes (including cytokines and chemokines), and with TLR4 being the only TLR family member who is able to activate NF-kB through two different signaling pathways [66]. The suppression of the TLR4 pathway is linked to worse outcomes and increased mortality in critically ill children with influenza [67] and the clearance of *S. pyogenes* in TLR4 knockout mice is significantly impaired [68], suggesting a key role of this gene in *S. pyogenes*-influenza coinfections.

### Transcriptional changes in the host common to *S. pyogenes* and IAV infections

In spite of the different mechanisms employed by *S. pyogenes* and IAV to adhere and infect the host cells, we found genes that were differentially expressed in host cells upon both infections. These included the DNA damage inducible transcript 4 (DDIT4), arrestin domain containing 3 (ARRDC3), the FosB proto oncogene (FOSB), and the URB2 ribosome biogenesis homolog. These genes are all related to the inflammatory response. First, DDIT4 (also known as REDD1/RTP801/Dig2) is a protein induced by different processes of cellular energy and stress, such as DNA damage, hypoxia and energy depletion, and regulates growth, proliferation and apoptosis through the inhibition of mTOR [69]. DDIT4 stimulates the expression of inflammatory genes in macrophages such as TNF-alpha and IL-1 beta [70], and its expression is increased upon *S. pneumoniae* infection [52] and after exposure to lipopolysaccharides (LPS) in different in vitro models [70, 71]. Conversely, DDIT4 knockdown and repression reduce vascular inflammation [70], reactive oxygen species generation triggered by LPS [71], and pro-inflammatory cytokines expression induced by *E.coli* infection [69]. Then, the arrestin domain containing 3 (ARRDC3) is part of the arrestin family, which regulate the signaling mediated by G-proteins, and as such, ARRDC3 is associated with cancer and inflammation. In the context of infections, ARRDC3 is increased in gastric epithelial cells by *H. pylori* infection in patients and animal models, and promotes gastric inflammation [72], and a ARRDC3 knockdown in HeLa cells reduces the susceptibility to the infection with the human papillomavirus (HPV; [73]). In turn, FOSB belongs to the Fos gene family of leucine zipper proteins that dimerize with the JUN family proteins and thus entails the AP-1 transcription factor complex. In this manner, and through AP-1, FOSB is associated with processes such as proliferation, differentiation, apoptosis, and the regulation of immunomodulatory genes [74]. FOSB expression is over twenty-fold and two-fold increased upon infections with *Neisseria gonorrhoeae* and *N. sicca*, respectively [75], and conditioned media from monocytes infected with *Mycobacterium tuberculosis* increased the expression of FOSB in astrocytes [76]. Finally, the URB2 gene encodes a protein conserved in mouse and yeast associated with ribosome biogenesis. URB2 is downregulated after rapamycin treatment, and is linked to processes of differentiation in the liver mediated by the mammalian target of rapamycin mTOR [77]. mTOR in turn is a kinase involved in multiple cellular functions related to homeostasis and inflammation, such as growth, proliferation, survival, protein synthesis and autophagy; and the rapamycin treatment reduces the inflammatory phenotype induced by mTOR [78].

Collectively, these four genes represent key regulatory nodes in cellular stress responses that connect mechanistically to both viral and bacterial pathogenicity. Among these, the upregulated genes DDIT4 and FOSB play central roles in cellular adaptation to infection. DDIT4 is a critical regulator of mTOR signaling and cellular metabolism under stress conditions [79, 80], while FOSB participates in immediate early transcriptional responses to cellular perturbation [81]. Both pathogens exploit these pathways to modulate host cell function for their replication. Specifically, disruption of mTOR signaling through DDIT4 upregulation affects multiple processes critical for pathogen survival including autophagy, protein synthesis, and immune responses [79, 82]. The cyclin-dependent kinase pathways targeted by our candidate compounds (flavopiridol and AT7519) regulate cell cycle progression and transcriptional control through RNA polymerase II phosphorylation. IAV hijacks cell cycle machinery to facilitate viral replication [83], while streptococcal infections induce cell cycle alterations as part of their pathogenic strategy [84]. Similarly, PLK1 inhibition by BI2536 affects both mitotic progression and inflammatory signaling pathways that are exploited by both pathogens [85, 86]. These mechanistic connections provide a computational and literature-supported rationale for why compounds targeting these host kinases may show activity against both viral and bacterial infections.

Moreover, we found 18 up- and 10 downregulated genes overlapping all three infections’ DEG sets before filtering for LFCs. In addition to the above-mentioned DDIT4, FOSB, ARRDC3 and URB2, other genes of interest include the upregulated mitochondrial genes (MT-ND4, MT-ND4L, MT-CYB, MT-CO1, MT-CO3, MT-ND2) and the transcription factors KLF6 and NFKBIZ, and the downregulated genes related to epigenetic control (HDAC9, H4C2, H4C13, H3C12), the two SLC20A1 homologs, the sialyltransferase ST3GAL1 and the integrin subunit alpha 2 (ITGA2). The downregulation of the cell adhesion gene ITGA2 is in agreement with former observations of reduced expression of integrins in IAV- infected cells [58], while changes in histone expression correlate with previous studies that show alterations in the chromatin structure upon IAV infection [87]. ST3GAL1 is a sialyltransferase whose expression is regulated by TGF beta and linked to cancer progression and chemoresistance, and while sialyltransferases are usually upregulated in cancer, ST3GAL1 display different patterns of up- and downregulation in various cell lines, and its overexpression increases cell migration [88]. Furthermore, besides the DEGs common to all three infections (DDIT4, FOSB, ARRDC3 and URB2), four other genes are also associated with inflammatory responses: KLF6, NFKBIZ, FST and TENT5A. The NFKBIZ inhibits the NF-kappa-B transcription factor complex activity, and hence controls inflammation and apoptosis [89], while the Krüppel-like factor 6 (KLF6) is a transcription factor involved in the regulation of several processes, such as differentiation, apoptosis and inflammation [90]; follistatin (FST) blocks the inflammatory response by binding the Activin A cytokine [91]; and TENT5A encodes a paralog of the nucleotidyltransferase TENT5C, a gene that increases the expression of immunoglobulins, which suggests that TENT5A may similarly regulate proteins secreted from B cells [92]. Taken together, our results from the expression analyses, the protein interactions obtained from STRINGdb, transcription binding sites found in the Harmonizome database, and evidences retrieved from the literature, indicate that these four genes are associated to the host response to both *S. pyogenes* and IAV infections through the AP-1 mediated signaling that regulates the gene expression of key-point genes involved in ECM composition, remodeling, and degradation.

### Candidate compounds to reverse the molecular phenotype during infection

In order to find compounds that could potentially reverse the molecular phenotypes caused by these infections, we applied a computational drug repurposing, using our gene expression data combined with information from drug intervention databases. Through this computational analysis, we identified the antitumoral and antibacterial compound mitoxantrone as well as three inhibitors of kinases, namely AT7519 and flavopiridol, that act against cyclin-dependent kinases (CDKs), enzymes involved in the regulation of the cell cycle also associated with transcription and apoptosis; and BI2536, an inhibitor of the Polo-like kinase 1 (PLK1), a conserved eukaryotic serine/threonine kinase with roles in the regulation of the cell cycle and division. Our results showed that flavopiridol is the top ranked drug to reverse the phenotypes during both *S. pyogenes* M1 and M49 infections when using data from perturbation databases (Table 1) but also had high interaction scores against IAV in our drug-target assessments (Table 2) and in the network analyses including genes common to all three infections (Table 3).

Mitoxantrone, an anticancer agent with wide spectrum antibacterial activity, is a synthetic anthracenedione, thus entailing an anthracycline structural core [93]. The antimicrobial activities of mitoxantrone have been studied in three separate high-throughput in vitro screenings against the Prestwick Chemical Library, a large collection of repurposed drugs containing over 1,200 compounds. First, mitoxantrone was found to enhance the antibacterial activity of colistin against planktonic *P. aeruginosa* [94]; another study showed that mitoxantrone potentiated the antibiotic tigecycline against *P. aeruginosa* [95]; and finally, mitoxantrone displayed antimicrobial activity against cultures of *S. pneumoniae*, by arresting its growth and decreasing the bacterial viability [93]. In addition, the antiviral effects of mitoxantrone have been shown for diverse types of viruses such as human herpes simplex virus HSV-1 [96], pseudorabies virus (PRV; [97]), human respiratory syncytial virus (HRSV; [98]) and the Rift Valley fever virus (RVFV; [99]).

AT7519 is a selective CDK inhibitor that induces apoptosis of neutrophils and eosinophils to promote the resolution of inflammations of the lung [100] and inflammations related to eosinophil-dominant allergies [101]. This resolution is also enhanced in the presence of bacterial infections, as shown in a model of *E.coli*- induced lung inflammation which also displayed reduced bacterial lung content upon AT7519 administration [102].

Flavopiridol (also known as alvocidib or HMR-1275) is a flavonoid alkaloid that targets various CDKs (initially attributed to CDKs 1, 2, 4, and 7) and later found to be most effective against CDK9, the catalytic subunit of the P-TEFb, a transcription elongation factor that acts as a positive regulator of mRNA transcription [103, 104]. In addition, CDK9 is associated with the control of the activity of the IAV RNA-dependent RNA polymerase [105]. Hence treating cells with flavopiridol not only blocks the mRNA synthesis which leads to apoptosis of the host cells, but also blocks the viral replication via the inhibition of the viral RNA polymerase [103, 105]. In this regard, a study showed the potent inhibitory activity of flavopiridol against several influenza types (H7N9, H3N2 and H1N1 including an antiviral-resistant variant of H1N1), with minimal cytotoxic effects in A549 cells [105]. For this, the authors screened a library of 273 kinase inhibitors in vitro. Moreover, Perwitasari et al. found that flavopiridol can be effectively administered together with other kinase inhibitors, acting synergistically, or in combination with neuraminidase inhibitors. Furthermore, a more recent study which involved a screening of 28 drugs potentially involved in recovering the cell viability upon influenza H5N1 infection showed that flavopiridol decreased the leukocyte infiltration and lung injury, thus improving the survival of H5N1-infected mice [106]. Thus flavopiridol is effective against infections with different IAV types both in vitro but also in vivo.

In turn, the peptide BI2536 is a highly specific inhibitor PLK1 that has been tested for its in vitro effects in human cells infected with IAV, both in cell lines (A549 cells) and tumor-free human lung cultures [107]. In these experiments, BI2536 induced inhibition of the early stages of infection in the A549 line, and a strong reduction of viral replication in the lung cultures with no toxicity observed.

Regarding prior evidence for our candidates in infectious disease contexts, BI2536 has demonstrated anti-IAV activity in vitro by inhibiting viral replication and reducing virus-induced cytopathic effects [107]. Flavopiridol similarly showed activity against IAV in cell-based models through its effects on cyclin-dependent kinases involved in viral transcription [106]. Mitoxantrone exhibits antibacterial activity including against Streptococcus pneumoniae [93], supporting its potential utility against streptococcal infections. AT7519, while lacking direct infectious disease validation, targets the same cyclin-dependent kinase pathways as flavopiridol, providing mechanistic rationale for its inclusion. In short, these previous studies validate our computational approach but also underscore that wet-lab validation of our specific predictions for *S. pyogenes*-IAV coinfections remains essential.

In addition, the toxicity profiles of our candidate compounds, while indicating caution is warranted, are comparable to many clinically used antimicrobials and do not preclude therapeutic development. Risk mitigation strategies include dose optimization through pharmacokinetic studies, targeted delivery approaches to minimize systemic exposure, and careful patient monitoring during clinical evaluation [45]. It should be noted that mitoxantrone and flavopiridol are already approved or in clinical trials for cancer treatment [108, 109], demonstrating that their toxicity profiles can be managed clinically. Furthermore, repurposing these compounds for acute infection treatment would likely involve shorter treatment durations than cancer therapy, potentially reducing cumulative toxicity concerns.

### Methodological considerations

A key methodological strength of our computational approach is the use of condition-specific analytical parameters that account for substantial differences in transcriptional response magnitudes across the three infection conditions. The M1 infection induced a focused transcriptional response with 84 DEGs, while M49 infection triggered a broader response with 1,650 DEGs, and IAV infection showed an intermediate profile. Rather than applying uniform read count filtering and fold-change thresholds across all conditions, we employed data-driven, adaptive thresholds appropriate to each biological context. This approach aligns with current best practices in RNA-seq analysis, which demonstrate that optimal filtering thresholds vary based on sequencing depth and experimental design, and that no single threshold maximizes statistical power across all scenarios [110, 111]. Recent benchmark studies have shown that uniform fold-change cutoffs can actually reduce inter-dataset reproducibility when applied to conditions with different biological response magnitudes [112], whereas adaptive threshold selection maintains false discovery rate control while maximizing detection power. Our condition-specific parameters ensured that identified DEGs represented robust transcriptional changes in each infection context, providing biologically meaningful input signatures for computational drug repositioning. The consistent statistical rigor applied across all analyses (adjusted p-value < 0.01) maintained comparable type I error control regardless of threshold differences, while the adaptive fold-change criteria prevented the M49 signature from overwhelming downstream analyses with an excessive number of weakly differential genes. This methodological approach facilitated the identification of shared host pathways across infections despite their markedly different transcriptional profiles, ultimately enabling the prediction of repositionable compounds with potential broad-spectrum activity against bacterial-viral coinfections.

### Limitations

Several limitations of our study should be acknowledged. Our reliance on a single pharyngeal epithelial cell line (Detroit 562) may not fully capture the complexity and heterogeneity of the respiratory epithelium responses during natural infections, where multiple cell and tissue types and immune interactions contribute to pathogenesis. In addition, while our dual RNA-seq approach captured host transcriptional responses, we focused exclusively on single-pathogen infections rather than actual coinfections due to both technical constraints in establishing appropriate bacterial controls and the challenges of recreating pathogen-pathogen-host interactions in vitro. This experimental design, while justified by the predominant role of host- mediated interactions in coinfection pathogenesis, represents a simplification of the clinical scenario, where pathogens may interact directly or sequentially modify host susceptibility.

From a translational perspective, our study represents a drug repurposing prediction rather than experimental validation of therapeutic efficacy. The four identified compounds—mitoxantrone, AT7519, flavopiridol, and BI2536—require rigorous experimental testing to confirm their ability to reverse infection-induced transcriptional signatures and demonstrate actual therapeutic benefit in relevant infection models. Furthermore, the translation from single-pathogen transcriptional signatures to effective coinfection therapeutics assumes that the shared host pathways we identified remain equally relevant and targetable during simultaneous or sequential pathogen infections, an assumption requiring empirical validation.

Specifically, the L1000CDS2 approach employed in this study predicts compounds based on their ability to reverse disease-associated gene expression signatures, but this does not guarantee therapeutic efficacy in biological systems [113]. Several factors not captured by transcriptomic analysis could affect compound performance including pharmacokinetics, tissue distribution, drug metabolism, and complex host-pathogen interactions [114]. Concordance between different connectivity mapping platforms is typically modest, as demonstrated in our validation analysis using iLINCS with original Connectivity Map signatures. While we observed drug class consistency, particularly for PLK1 and cyclin-dependent kinase inhibitors, only mitoxantrone appeared in both L1000CDS2 and Connectivity Map predictions for the same condition. This highlights that computational predictions should be viewed as hypothesis-generating tools rather than definitive evidence of therapeutic activity.

Beyond these methodological considerations, several critical questions remain: whether our compounds exert effects through general cytotoxicity rather than specific beneficial host modulation, whether targeting individual pathogens might prove more clinically effective than our dual-targeting approach, and the potential for negative interactions between predicted compounds or with standard antibiotics and antivirals. These considerations underscore the need for systematic wet-lab validation to transition from computational predictions to clinically viable therapeutics. Nevertheless, our computational approach identified novel drug candidates with demonstrated activity against related pathogens, providing a cost-effective foundation for future experimental validation and offering promising avenues for developing novel therapeutics for these challenging coinfections.

Furthermore, another key limitation of this study is the absence of experimental validation for the predicted drug candidates in *S. pyogenes*-IAV coinfection models. While our computational approach successfully identified compounds with mechanistic rationale and literature support in related infectious disease contexts, in vitro and in vivo validation remains essential to confirm therapeutic efficacy, determine optimal dosing regimens, and assess potential toxicity in the context of active coinfection. The L1000CDS2 connectivity mapping approach employed in this study predicts compounds based on their ability to reverse disease-associated gene expression signatures, but this does not guarantee therapeutic efficacy in biological systems [113]. Several factors not captured by transcriptomic analysis could affect compound performance, including pharmacokinetics, tissue distribution, drug metabolism, host immune interactions, and the complex spatial and temporal dynamics of coinfection pathogenesis [114]. Our cross-validation analysis using the iLINCS platform demonstrated drug class consistency, particularly for PLK1 and cyclin-dependent kinase inhibitors, but also revealed that exact compound concordance between different connectivity mapping databases is modest. This highlights that computational predictions should be viewed as hypothesis-generating tools that prioritize candidates for experimental testing rather than definitive evidence of therapeutic activity. Future studies should include dose-response assessment in relevant cell-based coinfection models, evaluation of compound effects on bacterial invasion and viral replication dynamics, assessment of host cell viability and immune response modulation, and ultimately validation in appropriate animal models of *S. pyogenes*-IAV coinfection. While BI2536 and flavopiridol have demonstrated anti-IAV activity in vitro [106, 107], and mitoxantrone shows antibacterial activity against *Streptococcus pneumoniae* [93], validation of these specific predictions for *S. pyogenes*-IAV coinfections represents essential future work. Nevertheless, our computational framework provides a cost-effective and time-efficient approach to prioritize repositionable drug candidates from the vast chemical space, offering a rational starting point for experimental validation programs aimed at addressing the urgent clinical need for improved therapeutics against severe bacterial-viral coinfections.

## Conclusions

In this study, we set out to computationally identify candidates for drug repurposing for the treatment of IAV-*S. pyogenes* coinfections. In vitro infection of Detroit 562 cells with either IAV or *S. pyogenes* allowed us to detect pathways, which were differentially expressed in the host under both conditions as a result of the host’s response. Since these genes were involved in the cellular stress response, inflammation, and apoptosis, the aim was to identify compounds that could reverse the molecular phenotype to treat the consequences of infection. Through computational drug repurposing, we were able to identify four compounds that have the potential to act as suitable candidate drugs for treating IAV-*S. pyogenes* coinfections pending experimental validation. The fact that two of our four top ranked drugs have been already proven to work against IAV infections in vitro (BI2536 and flavopiridol; [105, 107]), and another against *S. pneumoniae* (mitoxantrone; [93]) further support not only our findings but also validates our computational approach, which avoids using large in vitro and in vivo drug screenings to find suitable drug candidates.

## Methods

### Cultivation and propagation of virus, eukaryotic and prokaryotic cells

Detroit 562 were originally isolated from the pharynx of a female pharyngeal cancer patient [115], and obtained from CLS Cell Lines Service (Eppelheim, Germany) on April, 28th, 2013. These cells were cultivated in Dulbecco’s modified eagle medium (DMEM)/F12 (1:1) (Thermo Fisher Scientific, Schwerte, Germany) supplemented with 10% FBS at 37 °C and 5% CO2. GAS strain AP1 and strain 591 were maintained for up to 7 days on columbia agar plates with 5% sheep blood (BD, Heidelberg, Germany) at 4 °C and cultivated in Todd-Hewitt broth, supplemented with 0.4% yeast extract (THY) (Thermo Fisher Scientific, Wesel, Germany) at 37 °C and 5% CO2. IAV strain A/Bavaria/74/2009 (H1N1) was kindly provided by PD Dr. med. habil. Jürgen Stech (Friedrich-Loeffler-Institut, Germany). For virus propagation, four 75 cm² cell culture flasks of confluent MDCK-II (Merck, Darmstadt, Germany) cells were washed once with 1x PBS. Subsequently, flasks received 15 ml minimum essential medium eagle (Merck, Darmstadt, Germany) containing 2 mM L-glutamine, 0.2% BSA and 2 µg/ml TPCK-treated trypsin. 50 µl of virus stock (1-5 × 107 TCID50/ml) was added to each flask. After 3 days of incubation at 37 °C and 5% CO2, detached cells in the supernatant were removed via centrifugation. Supernatants were centrifuged at 20,000 rpm (MLA-80 rotor, 27,700 x g at rmax) and 4 °C for 4 h. Virus pellets were resuspended in 1x PBS and stored at -80 °C. 50% tissue culture infectivity dose (TCID50) was calculated using the Spearman and Kärber algorithm [116]. In brief, ten-fold serial dilutions of the virus were administered to confluent MDCK-II cells in octuplicates in the presence of TPCK-trypsin for 3 d at 37 °C, 5% CO2. Cell death was determined by visual inspection. For calculation, wells displaying a cytopathic effect were taken into account.

### Infection of detroit 562 cells with GAS

An overnight culture of GAS serotype M1 or serotype M49 was diluted 1:20 in pre-warmed THY and grown to OD_600 nm_= 0.4. Cultures were washed once with 1x PBS and resuspended in DMEM/F-12 + 10% FBS. Prior to infection, 24-well plates containing confluent Detroit 562 cells were washed twice with 1x PBS. 1 ml of bacterial suspension (MOI 100) or bacteria-free medium was added to washed cells & one empty well (B0). Cells and bacteria were incubated for 2 h at 37 °C, 5% CO_2_. After incubation, well plates were placed on ice and supernatant was collected. Detached cells were collected by centrifugation at 300 x *g* and 4 °C. Adherent cells were detached with a cell scraper, collected by centrifugation and combined with the cells from the supernatant. Pellets were washed once with pre-cooled 1x PBS, then shock-frozen in liquid nitrogen and stored at -20 °C. Supernatants were stored at -80 °C. Pellets intended for subsequent RNA-isolation were lysed with 350 µl Buffer RLT per 1 × 10^6^ cells from the RNeasy Mini Kit (Qiagen, Hilden, Germany). Lysates were homogenized by vortexing for 1 min, shock-frozen in liquid nitrogen and stored at -80 °C. For determination of GAS adherence to Detroit 562 cells, adherent cells were washed twice with 1x PBS and then detached by trypsin treatment. Detached cells were pelleted and lysed by resuspending in 1 ml sterile water. Suspensions were incubated for 10 min at RT with occasional vortexing. Serial dilutions of lysates were plated on THY agar to determine CFU/ml of adherent cells. CFU/ml of adherent cells were divided by total CFU/ml (B0) to calculate the percentage of bacterial adherence.

### Infection of detroit 562 cells with IAV

Detroit 562 cells were seeded into 24-well plates and grown to confluence. Cells were washed twice with 1x PBS. Subsequently, 500 PFU of IAV in 1 ml DMEM/F-12 containing, 0.2% BSA and 2 µg TPCK-treated trypsin (Merck, Darmstadt, Germany) was added. Uninfected cells received the identical medium without addition of 500 PFU of IAV. Cells and viruses co-incubated for 48 h at 37 °C, 5% CO_2_. After incubation, supernatants and pellets were collected as described above.

### Experimental controls

The experimental design included mock-infected control samples for each infection condition. Detroit 562 cells were treated with phosphate-buffered saline (PBS) for bacterial infection controls and with uninfected cell culture medium for IAV infection controls, collected at matched time points (2 h post-infection for bacterial controls, 48 h post-infection for viral controls).

### RNA isolation

RNA from 1.5 to 2 wells was isolated with the RNeasy Mini Kit according to the manual. 7.5 µg RNA were treated with 2 U of DNase I from the Turbo DNA-free kit (Thermo Fisher Scientific, Schwerte, Germany) according to the manufacturer’s instructions. RNA quantity was measured with the Qubit RNA HS Assay kit (Thermo Fisher Scientific, Schwerte, Germany) and a Qubit Fluorometer 2.0. RNA quality was assessed using a 2100 Bioanalyzer (Agilent, Waldbronn, Germany) and the RNA 6000 Nano kit (Agilent, Waldbronn, Germany). RNA samples with a RIN > 8.5 were deemed suitable for further analyses. RNA was stored at -80 °C until further use. Success of DNase treatment was controlled via qPCR using *hGAPDH*_RT_fwd (GCTGGCGCTGAGTACGTCGT) and *hGAPDH*_RT_rev (CCTGCAAATGAGCCCCAGCC) supplied by Eurogentec. RNA samples with Cq values > 30 were deemed to be DNA-free.

### cDNA synthesis and qPCR

500 ng of DNA-free RNA was reversely transcribed to cDNA using the SuperScript III First-Strand Synthesis SuperMix (Thermo Fisher Scientific, Schwerte, Germany) according to the manufacturer’s instructions. Synthesized cDNA was diluted 1:20 with DEPC-treated water.

Four µl of 1:20-diluted cDNA was used as template for qPCR. In addition, 20 µl qPCR reaction mix contained 0.75 µM fwd/rev primer and 1x Maxima SYBR Green/ROX 2x qPCR Master Mix (Thermo Fisher Scientific, Schwerte, Germany). 40 cycles were performed with annealing at 60 °C using the ViiA 7 Real-Time PCR system and software (Thermo Fisher Scientific, Schwerte, Germany). Only primers with an efficiency > 80% were used for qPCR. Relative transcript abundance was calculated employing the 2^−ΔΔCT^ method. The gene for human GAPDH served as reference gene.

### Enzyme-linked immunosorbent assay (ELISA)

Cytokine concentration in supernatants was determined with ELISA MAX Deluxe kits (BioLegend, Koblenz, Germany) according to the manufacturer’s instructions. In short, 96-well plates were covered with 1x Capture Antibody for 18 h at 4 °C. Subsequently, plates were washed several times with PBST. Non-specific binding was blocked by addition of 1x Assay Diluent A for 1 h at RT under constant agitation. After several wash steps with PBST, sample and standard were added for 2 h at RT under constant agitation. Samples and standards were loaded in duplicates. Following incubation, wells were washed as previously described and 1x Detection Antibody was added for 1 h at RT under constant agitation. Avidin-HRP was added for 30 min at RT under constant agitation after several wash steps. Lastly, wells were washed and Substrate Solution was added. Chromogenic reaction was stopped after 15–30 min depending on the kit. Absorbance was read at 450 nm with a VersaMax Microplate Reader (Molecular Devices, München, Germany).

### Trypan blue staining

Following infection with GAS, Detroit 562 cells were detached with 200 µl accutase. 200 µl 1x PBS was added and 25 µl of that cell suspension were mixed 1:1 with 0.4% Trypan Blue. 10 µl of the mixture was loaded into a counting chamber to determine the sample’s live/dead-ratio.

### SDS-PAGE and western blot analyses

Pellets collected from the infection model were covered with 50 µl RIPA Lysis and Extraction Buffer (Thermo Fisher Scientific, Schwerte, Germany). The mixture was agitated for 30 min at 4 °C. Subsequently, the suspension was centrifuged for 15 min at 14,000 x *g* and 4 °C to collect the raw cell lysate. The Pierce BCA Protein Assay Kit (Thermo Fisher Scientific, Schwerte, Germany) was utilized to measure protein concentration according to the manufacturer’s instructions. If necessary, a trichloroacetic acid (TCA) precipitation of protein samples preceded sample preparation for SDS-PAGE. One volume of 100% TCA was added to four volumes of protein sample. The mixture was incubated for 30 min at 4 °C and centrifuged for 5 min at 14,000 rpm and 4 °C afterwards. The pellet was covered with 500 µl cold acetone and centrifuged again. This step was repeated once. Overlay acetone was discarded and the pellet was dried by incubating at 95 °C for 5 min. The dried pellet was resuspended in 1x SDS-PAGE sample buffer. For SDS-PAGE analysis, 20 µg of whole cell lysate was mixed with SDS-PAGE sample buffer and loaded into a 12% polyacrylamid gel for electrophoresis. Afterwards, polyacrylamid gels were stained with Coomassie Blue G250 staining solution. A second unstained gel was subjected to Western Blotting onto a PVDF membrane using a Trans-Blot SD Semi-Dry Transfer Cell (Bio-Rad, Feldkirchen, Germany). After protein transfer, membranes incubated for at least 1 h in Odyssey Blocking Buffer (LI-COR, Bad Homburg, Germany) at 4 °C. Subsequently, 1:250-diluted Apoptosis Western Blot Cocktail (Abcam, Cambridge, UK) was added to the membrane for 1 h at RT under constant agitation. After 3 washing steps, a secondary antibody mix composed of IRDye 800CW goat anti-mouse and goat anti-rabbit IgG (LI-COR, Bad Homburg, Germany) was added at a 1:5000 dilution for 1 h at RT under constant agitation. Lastly, the membrane was washed and documented using an Odyssey Infrared Imager (LI-COR, Bad Homburg, Germany).

### Sequencing and bioinformatics

The reads were sequenced at Chronix Biomedicals on Illumina NextSeq 500 platform, as strand-specific (reverse-stranded) with single-end 75 bp reads (~ 30 M reads per sample). The raw reads were trimmed with Trimmomatic version 0.39 [117] ). Meanwhile, the reads’ quality was checked with FastQC version 0.11.8 [118] and MultiQC version 1.2 [119], before and after the trimming. The trimmed reads were mapped against the host genome with STAR version 2.7.1a [120]. Then, the un-mapped reads from the host were aligned against the rightful pathogen genome with STAR version 2.7.1a. GRCh38 and GRCh38.95 were used as the human reference genome and its annotation, respectively. On the other hand, ASM99376v1 (GenBank: GCA_000993765.1), ASM1812v1 (GenBank: GCA_000018125.1) and ViralMultiSegProj15622 (GenBank: not available) reference genomes were used for GAS serotype M1, GAS serotype M49 and IAV, respectively. The mapped reads (read counts per gene) table was prepared with the HTSeq-count version 0.6. Tool [121]. From this data, the Scotty tool [122] was used to measure the transcription depth and the gene’s coverage (p-value = 0.05 and FC = 2) of the generated count tables.

Prior to differential expression analysis, we applied infection-specific read count thresholds to remove genes with insufficient expression levels. These thresholds were established by examining the distribution of read counts in each dataset and identifying points where stable gene expression measurements could be reliably distinguished from background noise. For M1 infections, we applied a threshold of 400 total mapped reads across all samples (combining treatment and control samples), retaining 10,368 genes for differential expression analysis. For M49 infections, a higher threshold of 1,800 reads was necessary due to lower overall sequencing depth in this condition, retaining 6,068 genes. For IAV infections, an intermediate threshold of 600 reads was used, retaining 9,388 genes. This adaptive filtering approach aligns with current best practices in RNA-seq analysis, which demonstrate that optimal filtering thresholds vary based on sequencing depth and experimental design rather than following universal cutoffs [110, 111]. Uniform read count thresholds across conditions with different sequencing depths can introduce bias by allowing more lowly expressed genes to pass filtering in deeply sequenced samples while inappropriately excluding genes in samples with lower coverage. Our condition-specific thresholds account for these technical differences while maintaining consistent statistical rigor across all analyses. Power analysis using Scotty confirmed that the retained genes provided adequate statistical power for detecting biologically relevant expression changes at the observed sequencing depths. The filtered mapped reads were used to assess differential expression with the DESeq2 package version 1.40.2 [123]. For this analysis, we used a DESeq2 protocol with a parametric fit type and an adjusted p-value threshold of 0.01 (FDR < 0.01) applied consistently across all conditions to maintain comparable type I error control. Condition-specific log2 fold-change (LFC) thresholds were applied to reflect the biological reality that the three infection conditions induced vastly different magnitudes of transcriptional response. For M1 and M49 infections, genes were considered differentially expressed if they met the criteria of adjusted p-value less than 0.01 and absolute log2 fold change greater than 1.0. For IAV infections, an absolute log2 fold change threshold of 0.5 was applied given the intermediate transcriptional response magnitude. Additionally, for M1 infections, we applied a secondary filter with |LFC| > 0.25 for functional annotation analyses to capture the full range of transcriptional changes in this condition, which exhibited a markedly smaller number of differentially expressed genes (84 DEGs) compared to M49 infections (1,650 DEGs). These condition-specific fold-change thresholds reflect the biological reality that different infection contexts warrant different analytical parameters to appropriately capture biologically meaningful changes. Recent benchmark studies demonstrate that applying uniform fold-change cutoffs across conditions with different biological response magnitudes can reduce reproducibility and fail to account for context-dependent effect sizes [112]. Our data-driven threshold selection approach maximizes statistical power while maintaining false discovery rate control [110], ensuring that identified DEGs in each condition represent robust transcriptional changes suitable for downstream drug repositioning analysis rather than artifacts of arbitrary uniform thresholds. A principal component analysis (PCA) of counts normalized by the variance stabilizing transformation method was used to evaluate variation among the samples and to identify potential outliers, as implemented in DESeq2. Then, gene ontology and KEGG pathway enrichments were obtained from the sets of up- and downregulated genes with the gprofiler2 package version 0.2.3 [124], against the *H. sapiens* annotations (p-value < 0.05) and with the g: SCS correction method implemented within gprofiler2. The obtained annotations were summarized using the REVIGO server [125], and annotations from selected gene sets were extracted with the EnrichrKG web application [126]. The network analysis was performed with the STRINGdb and the Harmonizome databases [41, 127].

Drug repositioning predictions were generated using the L1000CDS2 server [42], which employs characteristic direction signature analysis to identify small molecules that can reverse disease gene expression signatures. For this, we used the up- and downregulated genes as inputs for L1000CDS2 to search for drugs that elicit antagonistic patterns of gene expression. Gene interaction network analyses were carried out with the STRING database, and the gene-compound interactions were displayed with the STITCH server version 5.0 [128] using high confidence interactions (score greater than 0.7).

To validate our drug repositioning predictions, we then performed cross-validation using the iLINCS platform (http://www.ilincs.org/ilincs/, accessed November 2025), which provides access to the original Connectivity Map (CMap) small molecule signatures. The iLINCS server compared our gene signatures against the CMap database (519 compounds) using connectivity scoring based on the Kolmogorov-Smirnov statistic [43]. Compounds were ranked by their z-scores, which indicate the strength and direction of the connectivity between the query signature and each compound’s expression signature. This analysis provided independent validation using a different reference database and algorithmic approach compared to the L1000CDS2 predictions. Complete iLINCS results for all three infection conditions are provided in Supplementary File 4.

Finally, toxicity predictions for the four candidate compounds (mitoxantrone, flavopiridol, AT7519, and BI2536) were generated using the ProTox-3 web server (https://tox.charite.de/protox3/, accessed November 2025) [46]. For each compound, we submitted the chemical structure and requested predictions for multiple toxicity endpoints including acute oral toxicity (LD50 values), hepatotoxicity, carcinogenicity, mutagenicity, cytotoxicity, immunotoxicity, and blood-brain barrier penetration. ProTox-3 employs machine learning models trained on experimental toxicity data to predict these endpoints based on molecular structure and physicochemical properties. Results were classified according to the Globally Harmonized System (GHS) toxicity classes, where class 1 represents fatal toxicity, class 2 fatal/lethal, class 3 toxic, class 4 harmful, class 5 may be harmful, and class 6 non-toxic. Complete toxicity prediction data for all compounds are provided in Supplementary File 3.

Code for the entire analytical workflow, including data preprocessing, differential expression analysis, functional enrichment, and preparation of input signatures for drug repositioning, is publicly available at https://gitlab.uni-rostock.de/wb283/bmcmicrobiol2026.

## Supplementary Information

Supplementary Material 1. Mapped read counts and differentially expressed genes.

Supplementary Material 2. Compounds predicted with L1000CDS2 and drug-gene interactions from STITCH.

Supplementary Material 3. Toxicity prediction data (ProTox-3).

Supplementary Material 4. Drug repositioning using iLINCS.

Supplementary Material 5: Supplementary Table 1. Mapping statistics of the RNA-seq libraries. Supplementary Table 2. Top 25 upregulated genes in M1 - infected cells. Supplementary Table 3. Top 25 downregulated genes in M1 – infected cells. Supplementary Table 4. Top 25 upregulated genes in M49 – infected cells. Supplementary Table 5. Top 25 downregulated genes M49. Supplementary Table 6. Differentially expressed genes common to both M1 and M49- infected cells. Supplementary Table 7. Gene ontology annotations of the differentially expressed genes (DEGs) shared between the M1 and M49- infected cells. Supplementary Table 8. Functional relatedness among the gene ontology annotations of the host DEGs in the three infections. Supplementary Table 9. Top 25 upregulated genes upon IAV infection. Supplementary Table 10. Top 25 downregulated genes upon IAV infection. Supplementary Table 11. Differentially expressed genes in all three infections. Supplementary Table 12. Gene ontology enrichments from the differentially expressed genes common to all three infections. Supplementary Table 13. Top 5 ranked compounds predicted for each infection. Supplementary Figure 1. Rarefaction curves and power optimization from the sequencing depth. Supplementary Figure 2. Significant gene ontologies in the three infections. Supplementary Figure 3. Interaction network of DEGs common to all infections. Supplementary Figure 4. Expression patterns of DEGs common to all infections before LFC filtering.

## Data Availability

The RNA-seq datasets generated and analyzed during the current study are publicly available in the European Nucleotide Archive (ENA) repository under accession number PRJEB46611 (https://www.ebi.ac.uk/ena/browser/view/PRJEB46611). Code for differential expression analysis, functional enrichment, and data processing are available in the GitLab repository at https://gitlab.uni-rostock.de/wb283/bmcmicrobiol2026.

## Abbreviations

IAV: Influenza A virus
MOI: Multiplicity of infection
DEGs: Differentially expressed genes
GAS: Group A Streptococcus
hpi: Hours post infection
PFU: Plaque forming units
LFC: log2-fold change
GO: Gene Ontology
BP: Biological Process
MF: Molecular Function
CC: Cellular Component
CDK: Cyclin-dependent kinase
PLK1: Polo-like kinase 1
LINCS: Library of Integrated Network-Based Cellular Signatures
qPCR: Quantitative polymerase chain reaction
ELISA: Enzyme-linked immunosorbent assay
