# Supplementary material for: Computational identification of novel therapeutic candidates for Streptococcus pyogenes and influenza A coinfections through transcriptomic-based drug repositioning
Source: BMC Microbiol. 2026 Apr 23;26:399. doi: 10.1186/s12866-026-05063-y (PMC13107719; doi:10.1186/s12866-026-05063-y)
Supplement: Supplementary file 5 — Supplementary Material 5: Supplementary Table 1. Mapping statistics of the RNA-seq libraries. Supplementary Table 2. Top 25 upregulated genes in M1 - infected cells. Supplementary Table 3. Top 25 downregulated genes in M1 – infected cells. Supplementary Table 4. Top 25 upregulated genes in M49 – infected cells. Supplementary Table 5. Top 25 downregulated genes M49. Supplementary Table 6. Differentially expressed genes common to both M1 and M49- infected cells. Supplementary Table 7. Gene ontology annotations of the differentially expressed genes (DEGs) shared between the M1 and M49- infected cells. Supplementary Table 8. Functional relatedness among the gene ontology annotations of the host DEGs in the three infections. Supplementary Table 9. Top 25 upregulated genes upon IAV infection. Supplementary Table 10. Top 25 downregulated genes upon IAV infection. Supplementary Table 11. Differentially expressed genes in all three infections. Supplementary Table 12. Gene ontology enrichments from the differentially expressed genes common to all three infections. Supplementary Table 13. Top 5 ranked compounds predicted for each infection. Supplementary Figure 1. Rarefaction curves and power optimization from the sequencing depth. Supplementary Figure 2. Significant gene ontologies in the three infections. Supplementary Figure 3. Interaction network of DEGs common to all infections. Supplementary Figure 4. Expression patterns of DEGs common to all infections before LFC filtering. [file 12866_2026_5063_MOESM5_ESM.docx]

Supplementary Table 1. Mapping statistics of the RNA-seq libraries

|  | Sequencing Reads  (Million) | Processed Reads  (Million) | Mapped  (Million) | Mapped to Host  (%) | Mapped to Pathogen  (%) |
| --- | --- | --- | --- | --- | --- |
| Detroit 562 | 32.81 | 32.74 | 32.16 | 98 | - |
| Detroit 562 / AP1 | 32.32 | 32.24 | 31.72 | 97.86 | 0.27 |
| Detroit 562 / 591 | 33.11 | 33.01 | 31.91 | 92.34 | 4.02 |
| Detroit 562 / IAV | 31.09 | 31 | 29.84 | 95.82 | 0.06 |

Supplementary Table 2. Top 25 upregulated genes in M1 - infected cells

| Accession | Name | Description | LFC | Padj |
| --- | --- | --- | --- | --- |
| ENSG00000186352 | ANKRD37 | ankyrin repeat domain 37 | 3.83 | 1.78E-49 |
| ENSG00000168209 | DDIT4 | DNA damage inducible transcript 4 | 3.02 | 1.00E-116 |
| ENSG00000113369 | ARRDC3 | arrestin domain containing 3 | 2.43 | 2.16E-50 |
| ENSG00000148926 | ADM | adrenomedullin | 2.42 | 2.45E-50 |
| ENSG00000167772 | ANGPTL4 | angiopoietin like 4 | 2.25 | 5.75E-07 |
| ENSG00000137331 | IER3 | immediate early response 3 | 1.52 | 1.15E-17 |
| ENSG00000182782 | HCAR2 | hydroxycarboxylic acid receptor 2 | 1.49 | 1.61E-03 |
| ENSG00000134107 | BHLHE40 | basic helix-loop-helix family member e40 | 1.48 | 2.02E-32 |
| ENSG00000112773 | TENT5A | terminal nucleotidyltransferase 5A | 1.45 | 1.57E-09 |
| ENSG00000199568 | RNU5A-1 | RNA, U5A small nuclear 1 | 1.32 | 6.69E-06 |
| ENSG00000211448 | DIO2 | iodothyronine deiodinase 2 | 1.27 | 2.08E-04 |
| ENSG00000127129 | EDN2 | endothelin 2 | 1.25 | 4.89E-06 |
| ENSG00000135245 | HILPDA | hypoxia inducible lipid droplet associated | 1.23 | 1.08E-04 |
| ENSG00000144802 | NFKBIZ | NFKB inhibitor zeta | 1.21 | 9.32E-04 |
| ENSG00000125740 | FOSB | FosB proto-oncogene, AP-1 transcription factor subunit | 1.18 | 3.95E-05 |
| ENSG00000198886 | MT-ND4 | mitochondrially encoded NADH:ubiquinone oxidoreductase core subunit 4 | 1.17 | 2.24E-11 |
| ENSG00000111981 | ULBP1 | UL16 binding protein 1 | 1.14 | 7.35E-03 |
| ENSG00000146674 | IGFBP3 | insulin like growth factor binding protein 3 | 1.14 | 4.56E-05 |
| ENSG00000212907 | MT-ND4L | mitochondrially encoded NADH:ubiquinone oxidoreductase core subunit 4L | 1.1 | 7.44E-10 |
| ENSG00000130600 | H19 | H19 imprinted maternally expressed transcript | 1.1 | 3.21E-03 |
| ENSG00000067082 | KLF6 | Kruppel like factor 6 | 1.09 | 1.56E-06 |
| ENSG00000248527 | MTATP6P1 | MT-ATP6 pseudogene 1 | 1.09 | 2.95E-10 |
| ENSG00000198899 | MT-ATP6 | mitochondrially encoded ATP synthase membrane subunit 6 | 1.08 | 2.61E-10 |
| ENSG00000175906 | ARL4D | ADP ribosylation factor like GTPase 4D | 1.05 | 4.42E-04 |
| ENSG00000107968 | MAP3K8 | mitogen-activated protein kinase kinase kinase 8 | 1.01 | 7.09E-04 |

Supplementary Table 3. Top 25 downregulated genes in M1 – infected cells

| Accession | Name | Description | LFC | Padj |
| --- | --- | --- | --- | --- |
| ENSG00000115844 | DLX2 | distal-less homeobox 2 | -1.4 | 7.30E-04 |
| ENSG00000175505 | CLCF1 | cardiotrophin like cytokine factor 1 | -1.4 | 3.78E-04 |
| ENSG00000103888 | CEMIP | cell migration inducing hyaluronidase 1 | -1.2 | 8.16E-06 |
| ENSG00000139318 | DUSP6 | dual specificity phosphatase 6 | -1.2 | 2.76E-06 |
| ENSG00000164949 | GEM | GTP binding protein overexpressed in skeletal muscle | -1.1 | 1.40E-04 |
| ENSG00000173334 | TRIB1 | tribbles pseudokinase 1 | -1 | 2.10E-06 |
| ENSG00000275221 | H2AC15 | H2A clustered histone 15 | -1 | 3.14E-03 |
| ENSG00000197153 | H3C12 | H3 clustered histone 12 | -1 | 4.51E-03 |
| ENSG00000158050 | DUSP2 | dual specificity phosphatase 2 | -1 | 9.82E-04 |
| ENSG00000101665 | SMAD7 | SMAD family member 7 | -0.9 | 3.04E-03 |
| ENSG00000139800 | ZIC5 | Zic family member 5 | -0.9 | 8.00E-03 |
| ENSG00000275126 | H4C13 | H4 clustered histone 13 | -0.9 | 2.08E-04 |
| ENSG00000278705 | H4C2 | H4 clustered histone 2 | -0.9 | 3.79E-03 |
| ENSG00000181143 | MUC16 | mucin 16, cell surface associated | -0.9 | 3.84E-04 |
| ENSG00000164626 | KCNK5 | potassium two pore domain channel subfamily K member 5 | -0.8 | 8.67E-03 |
| ENSG00000112159 | MDN1 | midasin AAA ATPase 1 | -0.8 | 1.14E-04 |
| ENSG00000196371 | FUT4 | fucosyltransferase 4 | -0.8 | 1.65E-03 |
| ENSG00000253729 | PRKDC | protein kinase, DNA-activated, catalytic subunit | -0.8 | 6.41E-03 |
| ENSG00000170385 | SLC30A1 | solute carrier family 30 member 1 | -0.8 | 1.05E-04 |
| ENSG00000144136 | SLC20A1 | solute carrier family 20 member 1 | -0.8 | 1.50E-03 |
| ENSG00000152804 | HHEX | hematopoietically expressed homeobox | -0.7 | 6.93E-03 |
| ENSG00000041982 | TNC | tenascin C | -0.7 | 5.94E-03 |
| ENSG00000164086 | DUSP7 | dual specificity phosphatase 7 | -0.7 | 1.97E-04 |
| ENSG00000008513 | ST3GAL1 | ST3 beta-galactoside alpha-2,3-sialyltransferase 1 | -0.6 | 3.72E-03 |
| ENSG00000048052 | HDAC9 | histone deacetylase 9 | -0.6 | 1.17E-03 |

Supplementary Table 4. Top 25 upregulated genes in M49 – infected cells

| Accession | Name | Description | LFC | Padj |
| --- | --- | --- | --- | --- |
| ENSG00000170345 | FOS | Fos proto-oncogene, AP-1 transcription factor subunit | 4.63 | 7.16E-155 |
| ENSG00000115414 | FN1 | fibronectin 1 | 3.74 | 6.40E-63 |
| ENSG00000120129 | DUSP1 | dual specificity phosphatase 1 | 3.3 | 1.10E-62 |
| ENSG00000144802 | NFKBIZ | NFKB inhibitor zeta | 3.18 | 3.68E-41 |
| ENSG00000023445 | BIRC3 | baculoviral IAP repeat containing 3 | 3 | 6.37E-32 |
| ENSG00000162772 | ATF3 | activating transcription factor 3 | 2.82 | 6.18E-24 |
| ENSG00000168209 | DDIT4 | DNA damage inducible transcript 4 | 2.8 | 1.44E-34 |
| ENSG00000130513 | GDF15 | growth differentiation factor 15 | 2.54 | 5.15E-33 |
| ENSG00000179094 | PER1 | period circadian regulator 1 | 2.54 | 1.10E-21 |
| ENSG00000175197 | DDIT3 | DNA damage inducible transcript 3 | 2.45 | 1.75E-19 |
| ENSG00000210082 | MT-RNR2 | mitochondrially encoded 16S rRNA | 2.43 | 6.22E-05 |
| ENSG00000087074 | PPP1R15A | protein phosphatase 1 regulatory subunit 15A | 2.4 | 1.12E-29 |
| ENSG00000067082 | KLF6 | Kruppel like factor 6 | 2.33 | 4.05E-15 |
| ENSG00000130766 | SESN2 | sestrin 2 | 2.3 | 5.90E-22 |
| ENSG00000120738 | EGR1 | early growth response 1 | 2.28 | 3.06E-22 |
| ENSG00000162366 | PDZK1IP1 | PDZK1 interacting protein 1 | 2.24 | 1.09E-13 |
| ENSG00000245532 | NEAT1 | nuclear paraspeckle assembly transcript 1 | 2.2 | 4.03E-06 |
| ENSG00000145113 | MUC4 | mucin 4, cell surface associated | 2.19 | 9.54E-18 |
| ENSG00000119950 | MXI1 | MAX interactor 1, dimerization protein | 2.17 | 3.65E-04 |
| ENSG00000198886 | MT-ND4 | mitochondrially encoded NADH:ubiquinone oxidoreductase core subunit 4 | 2.17 | 1.15E-37 |
| ENSG00000113369 | ARRDC3 | arrestin domain containing 3 | 2.15 | 2.80E-13 |
| ENSG00000212907 | MT-ND4L | mitochondrially encoded NADH:ubiquinone oxidoreductase core subunit 4L | 2.15 | 1.71E-35 |
| ENSG00000221869 | CEBPD | CCAAT enhancer binding protein delta | 2.11 | 1.53E-28 |
| ENSG00000111674 | ENO2 | enolase 2 | 2.11 | 3.39E-18 |
| ENSG00000157514 | TSC22D3 | TSC22 domain family member 3 | 2.08 | 7.57E-21 |

Supplementary Table 5. Top 25 downregulated genes M49

| Accession | Name | Description | LFC | Padj |
| --- | --- | --- | --- | --- |
| ENSG00000166670 | MMP10 | matrix metallopeptidase 10 | -3.46 | 1.21E-41 |
| ENSG00000172818 | OVOL1 | ovo like transcriptional repressor 1 | -2.81 | 9.81E-40 |
| ENSG00000170385 | SLC30A1 | solute carrier family 30 member 1 | -2.73 | 7.77E-39 |
| ENSG00000205420 | KRT6A | keratin 6A | -2.61 | 1.14E-26 |
| ENSG00000111696 | NT5DC3 | 5’-nucleotidase domain containing 3 | -2.27 | 1.44E-34 |
| ENSG00000128594 | LRRC4 | leucine rich repeat containing 4 | -2.2 | 4.36E-21 |
| ENSG00000148677 | ANKRD1 | ankyrin repeat domain 1 | -2.2 | 1.60E-16 |
| ENSG00000213626 | LBH | LBH regulator of WNT signaling pathway | -2.16 | 2.37E-27 |
| ENSG00000106278 | PTPRZ1 | protein tyrosine phosphatase receptor type Z1 | -2.16 | 2.98E-19 |
| ENSG00000149212 | SESN3 | sestrin 3 | -2.08 | 1.26E-24 |
| ENSG00000103888 | CEMIP | cell migration inducing hyaluronidase 1 | -2.08 | 2.12E-19 |
| ENSG00000134363 | FST | follistatin | -2.07 | 2.76E-17 |
| ENSG00000106003 | LFNG | LFNG O-fucosylpeptide 3-beta-N-acetylglucosaminyltransferase | -1.99 | 2.68E-04 |
| ENSG00000111859 | NEDD9 | neural precursor cell expressed, developmentally down-regulated 9 | -1.96 | 1.83E-28 |
| ENSG00000131746 | TNS4 | tensin 4 | -1.89 | 4.96E-15 |
| ENSG00000196611 | MMP1 | matrix metallopeptidase 1 | -1.89 | 9.61E-13 |
| ENSG00000179431 | FJX1 | four-jointed box kinase 1 | -1.8 | 7.22E-18 |
| ENSG00000168453 | HR | HR lysine demethylase and nuclear receptor corepressor | -1.79 | 7.44E-17 |
| ENSG00000157240 | FZD1 | frizzled class receptor 1 | -1.78 | 1.17E-22 |
| ENSG00000196754 | S100A2 | S100 calcium binding protein A2 | -1.78 | 1.63E-48 |
| ENSG00000086548 | CEACAM6 | CEA cell adhesion molecule 6 | -1.74 | 9.37E-04 |
| ENSG00000100413 | POLR3H | RNA polymerase III subunit H | -1.7 | 1.42E-08 |
| ENSG00000163513 | TGFBR2 | transforming growth factor beta receptor 2 | -1.67 | 1.35E-29 |
| ENSG00000148053 | NTRK2 | neurotrophic receptor tyrosine kinase 2 | -1.66 | 5.36E-14 |
| ENSG00000087494 | PTHLH | parathyroid hormone like hormone | -1.64 | 5.79E-20 |

Supplementary Table 6. Differentially expressed genes common to both M1 and M49- infected cells

| GeneID | Gene | Description | LFC  M1 | LFC  M49 |
| --- | --- | --- | --- | --- |
| ENSG00000168209 | DDIT4 | DNA damage inducible transcript 4 | 3.02 | 2.8 |
| ENSG00000113369 | ARRDC3 | arrestin domain containing 3 | 2.43 | 2.15 |
| ENSG00000148926 | ADM | adrenomedullin | 2.42 | 1.64 |
| ENSG00000127129 | EDN2 | endothelin 2 | 1.25 | -1.57 |
| ENSG00000144802 | NFKBIZ | NFKB inhibitor zeta | 1.21 | 3.18 |
| ENSG00000125740 | FOSB | FosB proto-oncogene, AP-1 transcription factor subunit | 1.18 | 1.75 |
| ENSG00000198886 | MT-ND4 | mitochondrially encoded NADH:ubiquinone oxidoreductase core subunit 4 | 1.17 | 2.17 |
| ENSG00000212907 | MT-ND4L | mitochondrially encoded NADH:ubiquinone oxidoreductase core subunit 4L | 1.1 | 2.15 |
| ENSG00000067082 | KLF6 | Kruppel like factor 6 | 1.09 | 2.33 |
| ENSG00000248527 | MTATP6P1 | MT-ATP6 pseudogene 1 | 1.09 | 1.9 |
| ENSG00000198899 | MT-ATP6 | mitochondrially encoded ATP synthase membrane subunit 6 | 1.08 | 1.81 |
| ENSG00000140465 | CYP1A1 | cytochrome P450 family 1 subfamily A member 1 | 1 | 1.53 |
| ENSG00000228253 | MT-ATP8 | mitochondrially encoded ATP synthase membrane subunit 8 | 0.98 | 1.9 |
| ENSG00000198804 | MT-CO1 | mitochondrially encoded cytochrome c oxidase I | 0.94 | 1.85 |
| ENSG00000198938 | MT-CO3 | mitochondrially encoded cytochrome c oxidase III | 0.92 | 1.53 |
| ENSG00000198888 | MT-ND1 | mitochondrially encoded NADH:ubiquinone oxidoreductase core subunit 1 | 0.91 | 2.08 |
| ENSG00000198712 | MT-CO2 | mitochondrially encoded cytochrome c oxidase II | 0.9 | 1.68 |
| ENSG00000198786 | MT-ND5 | mitochondrially encoded NADH:ubiquinone oxidoreductase core subunit 5 | 0.88 | 1.5 |
| ENSG00000198763 | MT-ND2 | mitochondrially encoded NADH:ubiquinone oxidoreductase core subunit 2 | 0.86 | 1.9 |
| ENSG00000225630 | MTND2P28 | MT-ND2 pseudogene 28 | 0.68 | 1.65 |
| ENSG00000134363 | FST | follistatin | -0.51 | -2.07 |
| ENSG00000135763 | URB2 | URB2 ribosome biogenesis homolog | -0.57 | -1.64 |
| ENSG00000170385 | SLC30A1 | solute carrier family 30 member 1 | -0.78 | -2.73 |
| ENSG00000103888 | CEMIP | cell migration inducing hyaluronidase 1 | -1.22 | -2.08 |

Supplementary Table 7 – Gene ontology annotations of the differentially expressed genes (DEGs) shared between the M1 and M49- infected cells.

| Accession | Source | Description | P-value |
| --- | --- | --- | --- |
| GO:0019646 | GO:BP | aerobic electron transport chain | 1.34E-10 |
| GO:0042773 | GO:BP | ATP synthesis coupled electron transport | 2.41E-10 |
| GO:0042775 | GO:BP | mitochondrial ATP synthesis coupled electron transport | 2.41E-10 |
| GO:0022904 | GO:BP | respiratory electron transport chain | 6.37E-10 |
| GO:0042776 | GO:BP | proton motive force-driven mitochondrial ATP synthesis | 3.57E-09 |
| GO:0015986 | GO:BP | proton motive force-driven ATP synthesis | 7.35E-09 |
| GO:0006754 | GO:BP | ATP biosynthetic process | 3.53E-08 |
| GO:0009145 | GO:BP | purine nucleoside triphosphate biosynthetic process | 4.11E-08 |
| GO:0009206 | GO:BP | purine ribonucleoside triphosphate biosynthetic process | 4.11E-08 |
| GO:0009201 | GO:BP | ribonucleoside triphosphate biosynthetic process | 5.96E-08 |
| GO:0006120 | GO:BP | mitochondrial electron transport, NADH to ubiquinone | 5.50E-06 |
| GO:0015990 | GO:BP | electron transport coupled proton transport | 1.28E-05 |
| GO:0015988 | GO:BP | energy coupled proton transmembrane transport, against electrochemical gradient | 1.28E-05 |
| GO:0032981 | GO:BP | mitochondrial respiratory chain complex I assembly | 1.62E-03 |
| GO:0010257 | GO:BP | NADH dehydrogenase complex assembly | 1.62E-03 |
| GO:0006123 | GO:BP | mitochondrial electron transport, cytochrome c to oxygen | 3.05E-03 |
| GO:0033108 | GO:BP | mitochondrial respiratory chain complex assembly | 9.26E-03 |
| GO:0015453 | GO:MF | oxidoreduction-driven active transmembrane transporter activity | 1.04E-12 |
| GO:0008137 | GO:MF | NADH dehydrogenase (ubiquinone) activity | 3.51E-07 |
| GO:0050136 | GO:MF | NADH dehydrogenase (quinone) activity | 3.98E-07 |
| GO:0003954 | GO:MF | NADH dehydrogenase activity | 4.50E-07 |
| GO:0003955 | GO:MF | NAD(P)H dehydrogenase (quinone) activity | 5.70E-07 |
| GO:0016655 | GO:MF | oxidoreductase activity, acting on NAD(P)H, quinone or similar compound as acceptor | 1.93E-06 |
| GO:0016651 | GO:MF | oxidoreductase activity, acting on NAD(P)H | 1.24E-05 |
| GO:0004129 | GO:MF | cytochrome-c oxidase activity | 3.25E-02 |
| GO:0016675 | GO:MF | oxidoreductase activity, acting on a heme group of donors | 3.25E-02 |
| GO:0046933 | GO:MF | proton-transporting ATP synthase activity, rotational mechanism | 4.99E-02 |

Supplementary Table 8 - Functional relatedness among the gene ontology annotations of the host DEGs in the three infections

|  | M1 - M49 | M1 - IAV | M49 - IAV |
| --- | --- | --- | --- |
| GO: Biological Process | 0.88 | 0.17 | 0.27 |
| GO: Molecular Function | 0.7 | 0 | 0 |
| GO: Cellular Component | 0.92 | 0.31 | 0.38 |

Supplementary Table 9. Top 25 upregulated genes upon IAV infection

| Accession | Name | Description | LFC | Padj |
| --- | --- | --- | --- | --- |
| ENSG00000163141 | BNIPL | BCL2 interacting protein like | 4.91 | 1.82E-40 |
| ENSG00000168447 | SCNN1B | sodium channel epithelial 1 subunit beta | 4.59 | 1.62E-34 |
| ENSG00000169474 | SPRR1A | small proline rich protein 1A | 4.58 | 3.29E-70 |
| ENSG00000169583 | CLIC3 | chloride intracellular channel 3 | 4.57 | 3.29E-70 |
| ENSG00000130592 | LSP1 | lymphocyte specific protein 1 | 4.53 | 3.93E-33 |
| ENSG00000143382 | ADAMTSL4 | ADAMTS like 4 | 4.42 | 1.34E-34 |
| ENSG00000188373 | C10orf99 | chromosome 10 open reading frame 99 | 4.38 | 5.77E-13 |
| ENSG00000110375 | UPK2 | uroplakin 2 | 4.33 | 1.82E-27 |
| ENSG00000140519 | RHCG | Rh family C glycoprotein | 4.33 | 5.19E-18 |
| ENSG00000143369 | ECM1 | extracellular matrix protein 1 | 4.28 | 2.69E-30 |
| ENSG00000169550 | MUC15 | mucin 15, cell surface associated | 4.28 | 5.87E-16 |
| ENSG00000016602 | CLCA4 | chloride channel accessory 4 | 4.25 | 3.52E-12 |
| ENSG00000073067 | CYP2W1 | cytochrome P450 family 2 subfamily W member 1 | 4.22 | 1.17E-22 |
| ENSG00000265972 | TXNIP | thioredoxin interacting protein | 4.21 | 4.86E-43 |
| ENSG00000130600 | H19 | H19 imprinted maternally expressed transcript | 4.15 | 2.01E-36 |
| ENSG00000166920 | C15orf48 | chromosome 15 open reading frame 48 | 4.15 | 2.64E-17 |
| ENSG00000100100 | PIK3IP1 | phosphoinositide-3-kinase interacting protein 1 | 4.14 | 7.34E-25 |
| ENSG00000175155 | YPEL2 | yippee like 2 | 4.06 | 2.07E-25 |
| ENSG00000167779 | IGFBP6 | insulin like growth factor binding protein 6 | 3.95 | 7.07E-39 |
| ENSG00000176046 | NUPR1 | nuclear protein 1, transcriptional regulator | 3.83 | 7.13E-16 |
| ENSG00000088002 | SULT2B1 | sulfotransferase family 2B member 1 | 3.82 | 1.23E-44 |
| ENSG00000127954 | STEAP4 | STEAP4 metalloreductase | 3.79 | 2.02E-22 |
| ENSG00000167757 | KLK11 | kallikrein related peptidase 11 | 3.79 | 7.31E-32 |
| ENSG00000149260 | CAPN5 | calpain 5 | 3.77 | 2.53E-31 |
| ENSG00000167653 | PSCA | prostate stem cell antigen | 3.74 | 3.11E-20 |

Supplementary Table 10. Top 25 downregulated genes upon IAV infection.

| Accession | Name | Description | LFC | Padj |
| --- | --- | --- | --- | --- |
| ENSG00000108691 | CCL2 | C-C motif chemokine ligand 2 | -6.9 | 1.11E-16 |
| ENSG00000118523 | CCN2 | cellular communication network factor 2 | -6.2 | 6.82E-255 |
| ENSG00000148677 | ANKRD1 | ankyrin repeat domain 1 | -5.8 | 9.73E-124 |
| ENSG00000078401 | EDN1 | endothelin 1 | -4.6 | 2.39E-57 |
| ENSG00000142871 | CCN1 | cellular communication network factor 1 | -4.6 | 0 |
| ENSG00000050730 | TNIP3 | TNFAIP3 interacting protein 3 | -4.3 | 7.62E-20 |
| ENSG00000111859 | NEDD9 | neural precursor cell expressed, developmentally down-regulated 9 | -3.8 | 9.16E-80 |
| ENSG00000136869 | TLR4 | toll like receptor 4 | -3.6 | 3.45E-21 |
| ENSG00000128342 | LIF | LIF interleukin 6 family cytokine | -3.5 | 4.97E-70 |
| ENSG00000074590 | NUAK1 | NUAK family kinase 1 | -3.4 | 1.12E-46 |
| ENSG00000137801 | THBS1 | thrombospondin 1 | -3.3 | 3.22E-11 |
| ENSG00000125740 | FOSB | FosB proto-oncogene, AP-1 transcription factor subunit | -3.3 | 7.51E-18 |
| ENSG00000170214 | ADRA1B | adrenoceptor alpha 1B | -3.2 | 2.15E-17 |
| ENSG00000082482 | KCNK2 | potassium two pore domain channel subfamily K member 2 | -3.2 | 4.67E-34 |
| ENSG00000273703 | H2BC14 | H2B clustered histone 14 | -3.2 | 1.65E-22 |
| ENSG00000213626 | LBH | LBH regulator of WNT signaling pathway | -3.1 | 7.91E-25 |
| ENSG00000072041 | SLC6A15 | solute carrier family 6 member 15 | -3.1 | 2.99E-34 |
| ENSG00000179431 | FJX1 | four-jointed box kinase 1 | -3.1 | 2.29E-35 |
| ENSG00000150551 | LYPD1 | LY6/PLAUR domain containing 1 | -3.1 | 1.90E-12 |
| ENSG00000013297 | CLDN11 | claudin 11 | -3.1 | 2.91E-14 |
| ENSG00000143942 | CHAC2 | ChaC glutathione specific gamma-glutamylcyclotransferase 2 | -3 | 5.89E-21 |
| ENSG00000232445 | EMSLR | E2F1 mRNA stabilizing lncRNA | -3 | 4.74E-22 |
| ENSG00000182103 | FAM181B | family with sequence similarity 181 member B | -3 | 7.89E-11 |
| ENSG00000164045 | CDC25A | cell division cycle 25A | -2.8 | 4.75E-33 |
| ENSG00000117877 | POLR1G | RNA polymerase I subunit G | -2.8 | 4.84E-25 |

Supplementary Table 11. Differentially expressed genes in all three infections

| Accession | Name | Description | M1 | M49 | IAV |
| --- | --- | --- | --- | --- | --- |
| ENSG00000168209 | DDIT4 | DNA damage inducible transcript 4 | 3.02 | 2.8 | 3.57 |
| ENSG00000113369 | ARRDC3 | arrestin domain containing 3 | 2.43 | 2.15 | 3.33 |
| ENSG00000134107 | BHLHE40 | basic helix-loop-helix family member e40 | 1.48 | 1.09 | 1.65 |
| ENSG00000112773 | TENT5A | terminal nucleotidyltransferase 5A | 1.45 | 0.99 | 2.13 |
| ENSG00000144802 | NFKBIZ | NFKB inhibitor zeta | 1.21 | 3.18 | 1.68 |
| ENSG00000125740 | FOSB | FosB proto-oncogene, AP-1 transcription factor subunit | 1.18 | 1.75 | -3.32 |
| ENSG00000198886 | MT-ND4 | mitochondrially encoded NADH:ubiquinone oxidoreductase core subunit 4 | 1.17 | 2.17 | 1.19 |
| ENSG00000146674 | IGFBP3 | insulin like growth factor binding protein 3 | 1.14 | 1.18 | 1.59 |
| ENSG00000212907 | MT-ND4L | mitochondrially encoded NADH:ubiquinone oxidoreductase core subunit 4L | 1.1 | 2.15 | 1.13 |
| ENSG00000067082 | KLF6 | Kruppel like factor 6 | 1.09 | 2.33 | 2.25 |
| ENSG00000198727 | MT-CYB | mitochondrially encoded cytochrome b | 0.95 | 1.39 | 1.04 |
| ENSG00000198804 | MT-CO1 | mitochondrially encoded cytochrome c oxidase I | 0.94 | 1.85 | 1.15 |
| ENSG00000198938 | MT-CO3 | mitochondrially encoded cytochrome c oxidase III | 0.92 | 1.53 | 0.8 |
| ENSG00000185650 | ZFP36L1 | ZFP36 ring finger protein like 1 | 0.89 | 1.21 | 2.1 |
| ENSG00000198763 | MT-ND2 | mitochondrially encoded NADH:ubiquinone oxidoreductase core subunit 2 | 0.86 | 1.9 | 0.75 |
| ENSG00000164442 | CITED2 | Cbp/p300 interacting transactivator with Glu/Asp rich carboxy-terminal domain 2 | 0.77 | 1.43 | 1.54 |
| ENSG00000115548 | KDM3A | lysine demethylase 3A | 0.75 | 1.34 | 2.07 |
| ENSG00000171940 | ZNF217 | zinc finger protein 217 | 0.61 | 0.99 | 0.78 |
| ENSG00000134363 | FST | follistatin | -0.51 | -2.07 | -1.75 |
| ENSG00000135763 | URB2 | URB2 ribosome biogenesis homolog | -0.57 | -1.64 | -2.69 |
| ENSG00000164171 | ITGA2 | integrin subunit alpha 2 | -0.57 | -1.06 | -0.71 |
| ENSG00000048052 | HDAC9 | histone deacetylase 9 | -0.6 | -0.49 | -0.96 |
| ENSG00000008513 | ST3GAL1 | ST3 beta-galactoside alpha-2,3-sialyltransferase 1 | -0.63 | -0.91 | -1.15 |
| ENSG00000144136 | SLC20A1 | solute carrier family 20 member 1 | -0.77 | -1.21 | -2.1 |
| ENSG00000170385 | SLC30A1 | solute carrier family 30 member 1 | -0.78 | -2.73 | -0.93 |
| ENSG00000278705 | H4C2 | H4 clustered histone 2 | -0.86 | -1.22 | -1.58 |
| ENSG00000275126 | H4C13 | H4 clustered histone 13 | -0.86 | -1.43 | -1.69 |
| ENSG00000197153 | H3C12 | H3 clustered histone 12 | -0.98 | -1.45 | -2.41 |

Supplementary Table 12. Gene ontology enrichments from the differentially expressed genes common to all three infections

| **group** | **aspect** | **term id** | **term name** | **adj. p-value** |
| --- | --- | --- | --- | --- |
| up | MF | GO:0009055 | electron transfer activity | 8.87E-08 |
| up | MF | GO:0022853 | active monoatomic ion transmembrane transporter activity | 1.09E-07 |
| up | MF | GO:0015399 | primary active transmembrane transporter activity | 8.93E-07 |
| up | MF | GO:0015078 | proton transmembrane transporter activity | 1.71E-06 |
| up | MF | GO:0022804 | active transmembrane transporter activity | 3.51E-04 |
| up | MF | GO:0016491 | oxidoreductase activity | 3.83E-04 |
| up | MF | GO:0022890 | inorganic cation transmembrane transporter activity | 4.10E-03 |
| up | MF | GO:0008324 | monoatomic cation transmembrane transporter activity | 5.57E-03 |
| up | MF | GO:0015318 | inorganic molecular entity transmembrane transporter activity | 1.04E-02 |
| up | MF | GO:0015075 | monoatomic ion transmembrane transporter activity | 1.36E-02 |
| up | BP | GO:0022904 | respiratory electron transport chain | 4.07E-07 |
| up | BP | GO:0022900 | electron transport chain | 7.08E-07 |
| up | BP | GO:0006119 | oxidative phosphorylation | 1.73E-06 |
| up | BP | GO:0001666 | response to hypoxia | 2.98E-06 |
| up | BP | GO:0036293 | response to decreased oxygen levels | 4.07E-06 |
| up | BP | GO:0070482 | response to oxygen levels | 7.34E-06 |
| up | BP | GO:1902600 | proton transmembrane transport | 8.43E-06 |
| up | BP | GO:0009060 | aerobic respiration | 1.11E-05 |
| up | BP | GO:0045333 | cellular respiration | 3.58E-05 |
| up | BP | GO:0006091 | generation of precursor metabolites and energy | 7.05E-05 |
| up | BP | GO:0015980 | energy derivation by oxidation of organic compounds | 2.92E-04 |
| up | BP | GO:0009628 | response to abiotic stimulus | 1.72E-03 |
| up | BP | GO:0046034 | ATP metabolic process | 4.29E-02 |
| down | MF | GO:0030527 | structural constituent of chromatin | 1.67E-03 |
| down | BP | GO:0006334 | nucleosome assembly | 2.34E-02 |
| down | BP | GO:0034728 | nucleosome organization | 3.73E-02 |

Supplementary Table 13 - Top 5 ranked compounds predicted for each infection.

| Broad Id | Compound | Rank | Infection |
| --- | --- | --- | --- |
| BRD-K79090631 | CGP-60474 | 1 | M1 |
| BRD-K43389698 | BMS-387032 | 2 | M1 |
| BRD-A60245366 | AS-601245 | 3 | M1 |
| BRD-K13390322 | AT-7519 | 4 | M1 |
| BRD-K43389698 | BMS-387032 | 5 | M1 |
| BRD-K87909389 | alvocidib (flavopiridol) | 1 | M49 |
| BRD-M16762496 | S1205 | 2 | M49 |
| BRD-A52530684 | Doxorubicin | 3 | M49 |
| BRD-K88560311 | AG-014699 (PF-01367338) | 4 | M49 |
| BRD-A11702965 | 230752 | 5 | M49 |
| BRD-K36363294 | I-BET151 | 1 | IAV |
| BRD-K44100512 | KIN001-043 | 2 | IAV |
| BRD-K76401790 | JNK-IN-5A | 3 | IAV |
| BRD-K11267252 | CH5424802 | 4 | IAV |
| BRD-K04853698 | LDN-193189 | 5 | IAV |

Supplementary Figure 1. Rarefaction curves and power optimization from the sequencing depth


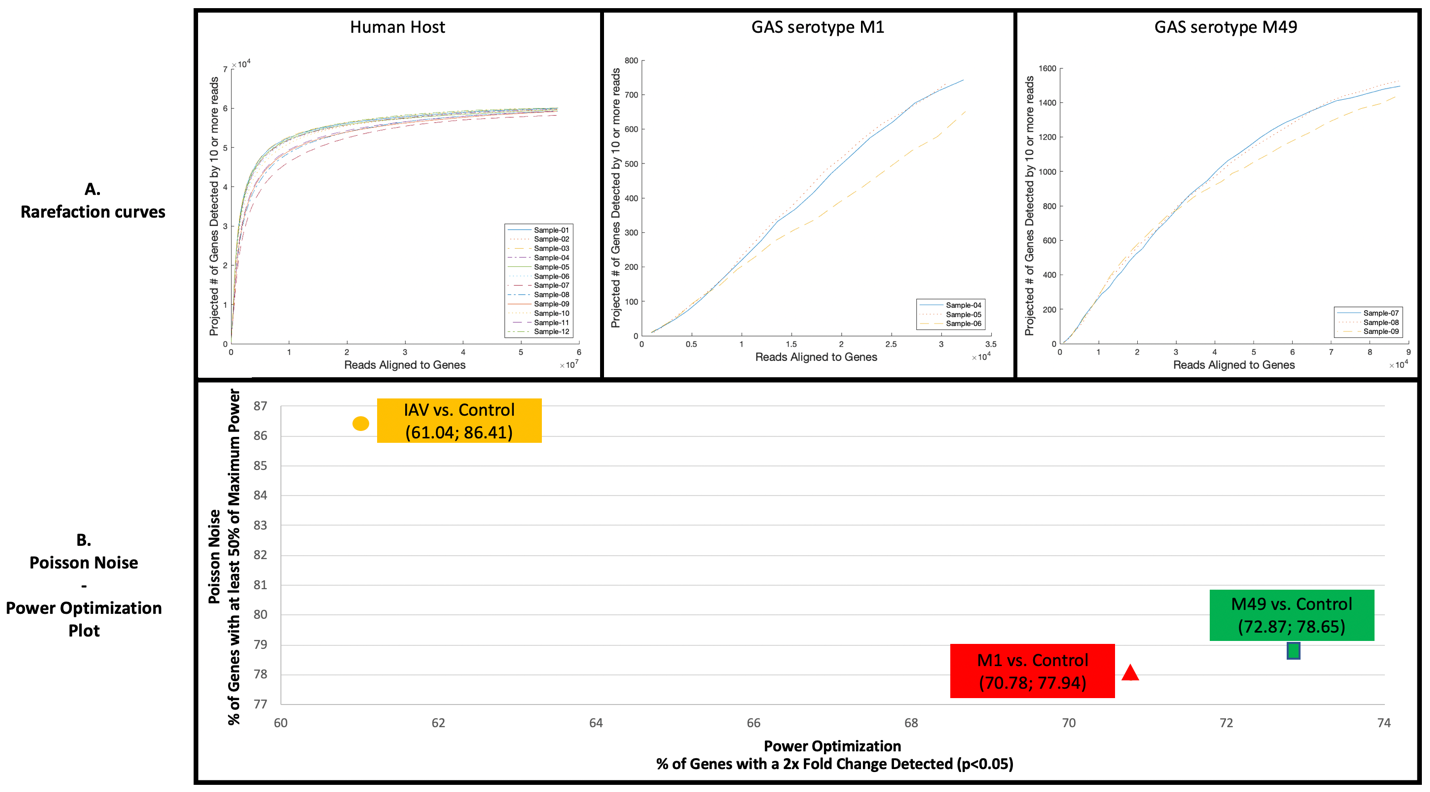


**(A) Rarefaction curves.** The rarefaction curves show how many genes are detected with 10 or more reads at each sequencing depth. Human host reads and reads from GAS serotypes M1 and M49, respectively, are plotted. **(B) Poisson Noise – Power Optimization plot.** Scotty’s Power plot and Poisson noise plot were analysed at 2x Fold Change and p<0.05. The working conditions of our experimental setup is 3 replicates with an average of 8.74M of reads aligned to genes per replicate.

Supplementary Figure 2. Significant gene ontologies in the three infections.


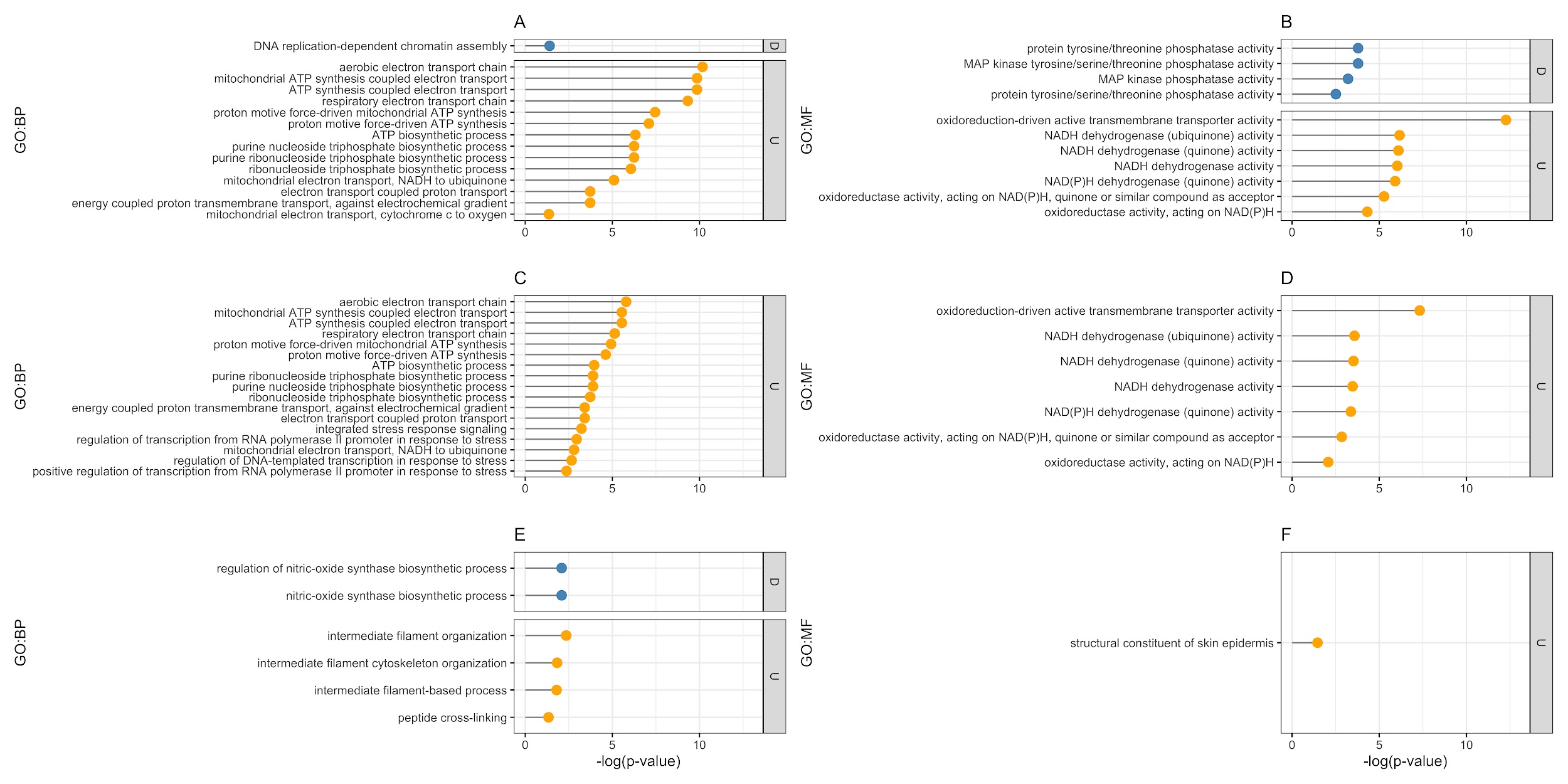


The plots correspond to the significant gene ontology biological process (GO:BP) and molecular function (GO:MF) annotations of the differentially expressed genes from the M1- infected (A and B), M49- infected (C and D), and IAV- infected cells (E and F), respectively, as calculated with gprofiler2 [117]. Descriptions of the significant ontologies are indicated in the *y*-axis, and their significance are shown in the *x*-axis as the *-log(p-value)*, with orange for the up- and blue for the downregulated annotations.

Supplementary Figure 3. Interaction network of DEGs common to all infections


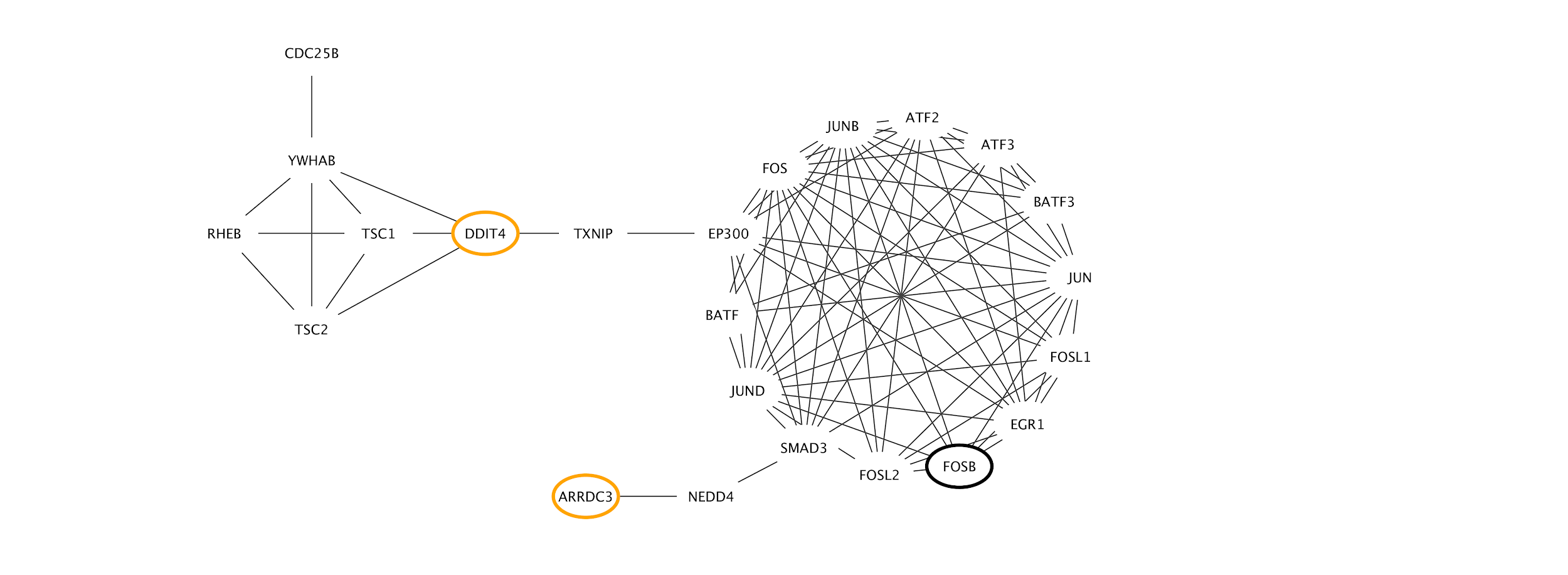


High confidence interactions (minimum required interaction score > 0.7) are displayed for genes interacting with those found as DEGs in the three infections: ARRDC3, DDIT4, FOSB and URB2. URB2 is not featured because it does not interact with these genes under this score threshold. Node border colors indicate upregulation in all three infections (orange), or displaying up- or downregulation in different infections (thick black border). Genes not found as DEGs in all infections but predicted as interacting are shown as nodes without borders. The interaction network was obtained with STRINGdb [106] (accessed 2023-02-07).

Supplementary Figure 4. Expression patterns of DEGs common to all infections before LFC filtering.


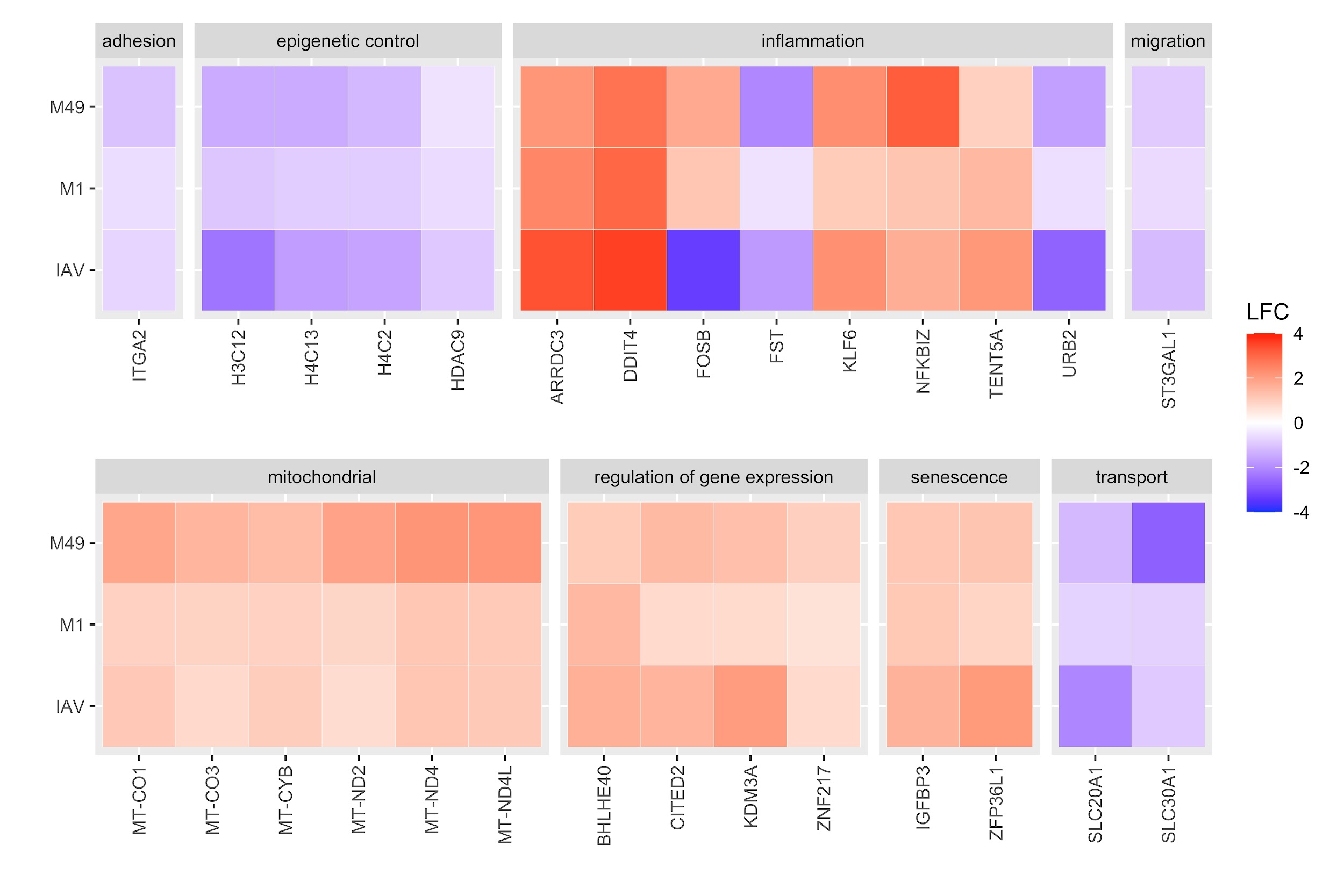


Heatmap showing the expression levels as log2- fold changes (LFC; respect to the control samples) of differentially expressed genes (DEGs) common to all three infections before applying the LFC- thresholds for each DEG set (Supplementary Table 11). Genes are stratified according to their corresponding annotations.

# 
